# Plasmalogens, the Vinyl Ether-Linked Glycerophospholipids, Enhance Learning and Memory by Regulating Brain-Derived Neurotrophic Factor

**DOI:** 10.3389/fcell.2022.828282

**Published:** 2022-02-09

**Authors:** Md. Shamim Hossain, Shiro Mawatari, Takehiko Fujino

**Affiliations:** Institute of Rheological Functions of Food, Fukuoka, Japan

**Keywords:** plasmalogens, memory, BDNF, synaptic plasticity, lipid raft, long-term potentiation (LTP), dendritic spine density

## Abstract

Plasmalogens (Pls), a kind of glycerophospholipids, have shown potent biological effects but their role in hippocampus-dependent memory remained mostly elusive. Here, we first report Pls can enhance endogenous expression of brain-derived neurotrophic factor (*Bdnf*) in the hippocampus and promotes neurogenesis associated with improvement of learning and memory in mice. Genomic and proteomic studies revealed that Pls enhanced recruitment of CREB transcription factor onto the murine *Bdnf* promoter region via upregulating ERK-Akt signaling pathways in neuronal cells. Reduction of endogenous Pls in murine hippocampus significantly reduced learning and memory associated with the reduction of memory-related protein expression, suggesting that Pls can regulate memory-related gene expression in the hippocampus.

## Introduction

Plasmalogens (PLs) are ether-linked glycerophospholipids that contain a vinyl ether bond at the *sn*-1 position of the glycerol moiety and consist of two main types, ethanolamine Pls (Pls-Etn) and choline Pls (Pls-Cho) (Braverman and Moser, 2012). The Pls-Etn are enriched mostly in the brain whereas the Pls-Cho are mostly abundant in other tissues including the heart and kidney (Braverman and Moser, 2012). It has been reported that up to two-thirds of the Etn-phospholipids in the whole brain is Pls-Etn (Nagan and Zoeller, 2001). Reduction of Pls-Etn in the brain and blood samples of Alzheimer’s disease (AD) patients and its association with the cognitive decline (Ginsberg et al., 1995; Wood et al., 2010; Braverman and Moser, 2012) has raised the question of whether there is a direct relation between Pls and the hippocampal-dependent memory.

Genetic mutation of two major peroxisomal enzymes related to Pls synthesizing enzymes, glyceronephosphate O-acyltransferase (GNPAT) and alkylglycerone phosphate synthase (AGPS), and their modulator Pex-7 has been found in Rhizomelic Chondrodysplasia Punctata (RCDP), which is a disorder characterized by developmental problems, congenital contractures, and severe intellectual disability (Braverman and Moser, 2012; Itzkovitz et al., 2012). The knockout mice of *AGPS* are lethal and those of *Pex-7* and *GNPAT* showed eye cataracts (Braverman and Moser, 2012; da Silva et al., 2014; Liegel et al., 2014) giving a difficulty to use these Pls-reduced mice models for a memory test. Due to the limitation of using the knockout mice, we employed a lentivirus system (sh-RNA) to knock down *GNPAT* mRNA expression, aiming to reduce local Pls content in the mouse hippocampus.

Our previous study showed that direct application of Pls activated cellular survival signaling of Akt and ERK1/2, resulting in the inhibition of neuronal cells death (Hossain et al., 2013). Consistent with our report, *Pex-7* and *GNPAT* knockout mice showed a deficit in Akt signaling in the peripheral Schwann cells, resulting in the development and differentiation problem of the myelination (da Silva et al., 2014). Our recent study showed that the Pls-induced activation of Akt and ERK signaling might be mediated by specific orphan G-protein coupled receptors (GPCR) expressed in neurons (Hossain et al., 2016). These studies indicate that Pls may act as mediators of cellular signaling in the nervous system.

BDNF is well known to regulate memory-related changes in the brain such as adult neurogenesis, synaptic protein expression, dendritic spine maturation, and synaptic plasticity including long-term potentiation (LTP) (Yamada and Nabeshima, 2003; Binder and Scharfman, 2004). It has been reported that BDNF can recruit TrkB, a target receptor of BDNF, into the membrane microdomains called lipid rafts to induce cellular signaling (Suzuki et al., 2004). Here, we studied the effects of Pls in the memory process and examined its regulating effects on BDNF-TrkB signaling. We also investigated whether oral ingestion of Pls could improve learning and memory in mice.

## Results

### Reduction of Hippocampal Plasmalogens Promoted Learning and Memory Loss in Adult Male Mice

**FIGURE 1:**
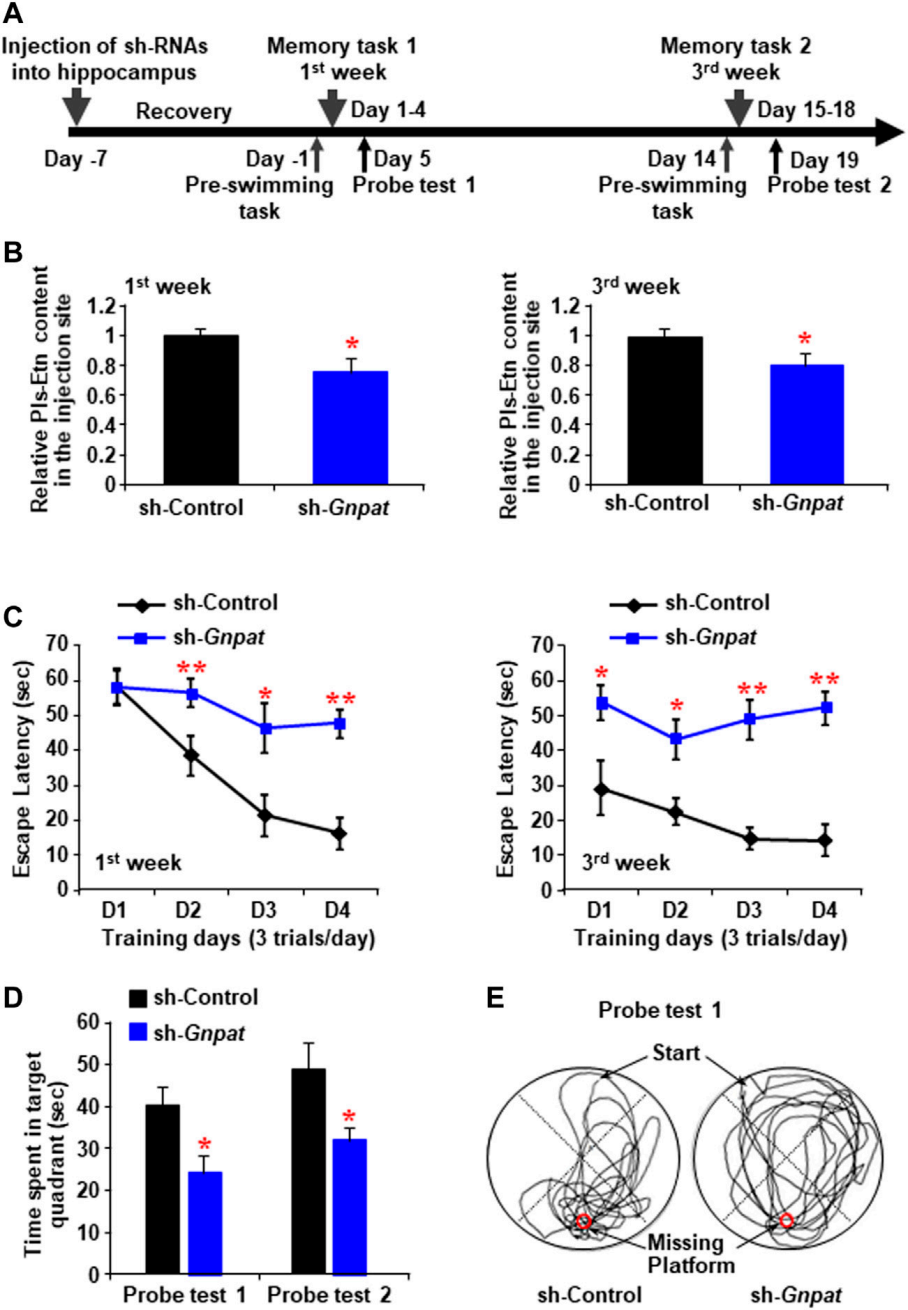
Memory impairment by the reduction of plasmalogen in the hippocampus. **(A)** Schematic diagram shows the memory tasks and probe trials schedule after the intrahippocampal injection of sh-RNA against *Gnpat*. **(B)** Mass spectroscopic data show the relative amount of ethanolamine Pls (Pls-Etn) in the hippocampus infected with lentiviruses (5 × 10^5^ TDU). **(C)** Morris water maze task shows in the change in escape latency at the first week (left panel) and third week (right panel). Data are the mean ± S.E.M. of three independent experiments. Each experiment included more than five mice whose hippocampus was successfully infected by sh-*GNPAT*. **(D)** Probe tests showed the time spent in the target quadrant. **(E)** Examples of traces for control (left) and sh-*GNPAT* (right) mice in probe test one performed after memory task 1. The red circle shows the place of the missing platform. *, *p* < 0.05 and **, *p* < 0.01, in each experimental group compared with the respective control group were analyzed by ANOVA test followed by post hoc Bonferroni’s tests.

To reduce the endogenous Pls synthesis, we injected lentiviral shRNA to knockdown *GNPAT* expression bilaterally into the mouse hippocampus (Figure 1A). Morris water maze tasks were performed following 1 (Memory task 1) and 3 weeks (Memory task 2) of the injection, each of which was followed by a probe test (Figure 1A). Reduction of brain Pls in the sh-*GNPAT* injected hippocampus was confirmed by liquid chromatography-mass spectrometry (LC-MS) analysis (Figure 1B). At both time points, significant increases in escape latency were observed in the sh-*GNPAT* group of mice compared with the control lentiviral (sh-Luciferase) group (Figure 1C). These data suggest that reduction of the hippocampal Pls reduced the learning performance in the mice. To assess the memory performance, we performed the probe tests after finishing the training phases. Probe tests showed that there was a significant reduction of the memory in mice groups lacking hippocampal Pls (Figures 1D,E). These findings suggest that the reduction of hippocampal Pls causes impairment of spatial learning and memory in mice. Control lentiviral injection showed no significant changes in the memory performance compared with the uninfected mice (Supplementary Figure S1A), indicating that the hippocampal injection itself did not disturb the spatial memory. In addition, the swimming speed of the mice was not affected by the lentivirus injection (Supplementary Figure S1B), suggesting that the decrease in learning and memory process in the sh-*GNPAT* mice was not associated with the motor activities.

### Knockdown of Hippocampal Plasmalogens Reduced the Memory-Related Gene Expression

**FIGURE 2:**
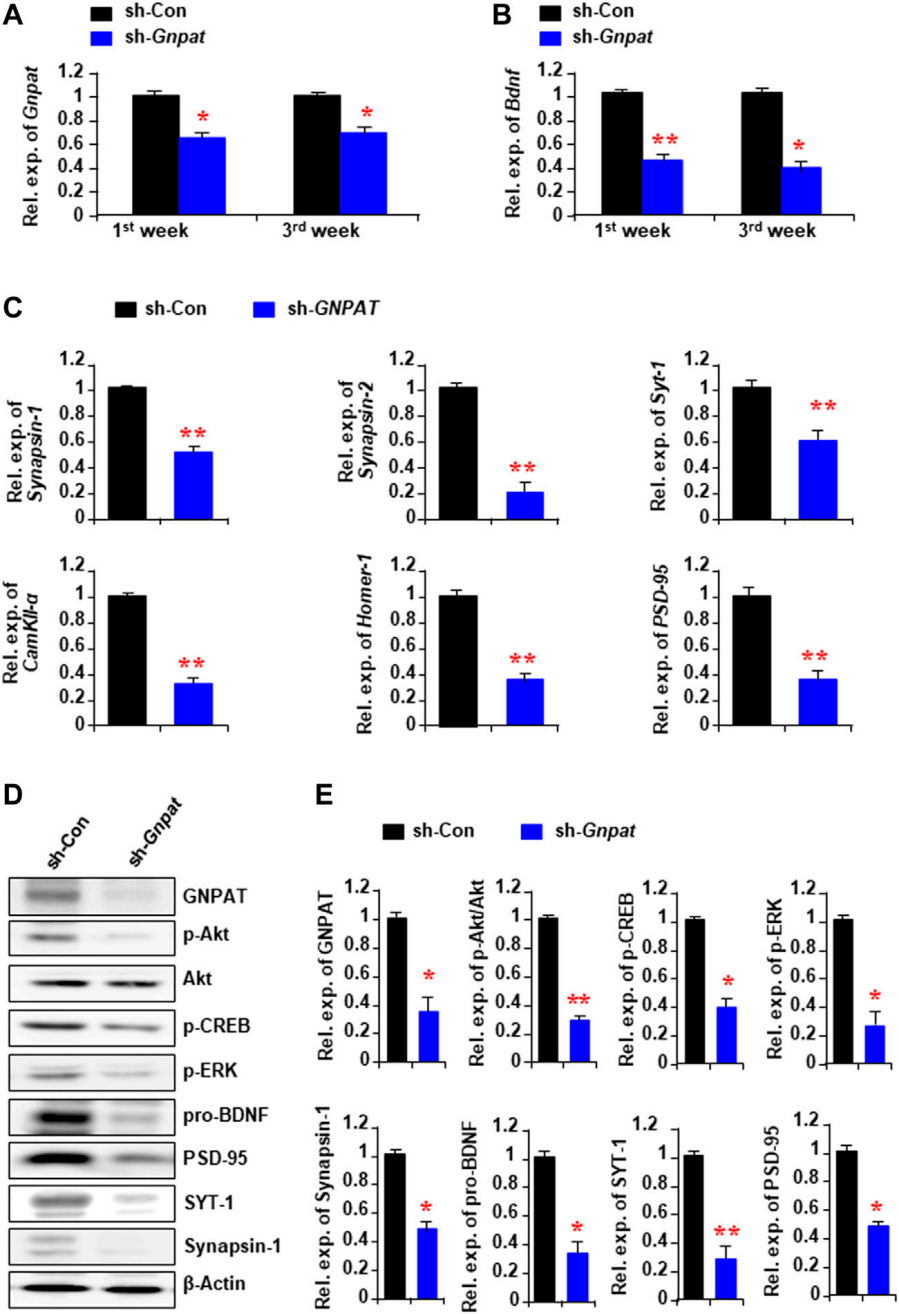
Alteration of memory-related gene expression and cellular signaling in the hippocampus following the Gnpat knockdown. **(A,B)** Real-time PCR data show the changes of *Gnpat* **(A)** and *Bdnf* **(B)** mRNA expressions in the hippocampus following 1 and 3 weeks after microinjection of sh-*Gnpat* lentiviruses. **(C)** Real-time PCR data show the changes in memory-related gene expressions in the hippocampus 3 weeks after the injections of lentiviruses. **(D)** Western blotting assays show the representative protein expressions in the hippocampus 3 weeks after the sh-control and sh-*Gnpat* injection. **(E)** Quantification data of panel **(D)** show the relative change of GNPAT, p-Akt, p-CREB, p-ERK, pro-BDNF, Synapsin-1, SYT-1, and PSD-95 in the hippocampus. The protein expression was normalized with the endogenous expression of β-Actin. The data represent the mean ± S.E.M. Each experiment included more than five mice. *, *p* < 0.05 and **, *p* < 0.01. The *p* values were obtained by Student’s t-tests.

To find out the cause of memory loss by the reduction of Pls in the hippocampus, we performed real-time PCR analysis with the infected hippocampal tissue samples, which showed a reduction of *GNPAT* mRNA expression both 1 and 3 weeks after the sh-*GNPAT* injection (Figure 2A). BDNF, one of the major memory-related neurotrophic factors, showed a significant reduction of its mRNA expression in the sh-*GNPAT* infected hippocampus (Figure 2B). Quantitative PCR data also showed a significant reduction of other memory-related gene expressions; e.g., *synapsin-1*, *synapsin-2*, *synaptotagmin-1* (*SYT-1*), *Ca* ^*2+*^ */calmodulin-dependent protein kinase II-α* (*CamKII-α*), *Homer-1*, and *PSD-95* in the Pls reduced hippocampus (Figure 2C).

We have previously found that the extracellular addition of Pls can activate ERK and Akt signaling in neuronal cells (Hossain et al., 2013). To examine the relationship between the reduction of Pls and the signaling of these kinases in the hippocampus, we performed Western blotting assays and found a significant reduction of phosphorylated proteins of ERK and Akt in the sh-*GNPAT* infected brain compared with the control group (Figure 2D). Protein expression of BDNF, synapsin-1, SYT-,1, and PSD95 was also found to be reduced in the knockdown tissues (Figures 2D,E) consistent with the mRNA expression data (Figures 2B,C). To confirm the regulatory effects of ERK and Akt phosphorylation on the expression of BDNF, synapsin-1, and SYT-1, we injected either ERK (U0126, 20 μM) or PI3K/Akt inhibitor (LY294002, 0.19 nmol) into the hippocampus (0.5 µL of 50 μM solution/site) stereotaxically in normal mice. It has been reported that these doses of U0126 and LY294002 were effective in blocking the signaling pathways in the brain tissues (Arroyo et al., 2018; Wang et al., 2018). Twenty-four hours after the injection we found a significant decrease in the mRNA expression of *Bdnf*, *synapsin-1,* and *SYT-1* in the hippocampus (Supplementary Figure S2). We, therefore, suggest that the reduction of the memory-related gene expression could be due to the down-regulation of ERK and Akt signaling pathways by the reduction of Pls in the hippocampus tissues.

### Plasmalogens Diet Enhances Memory and Memory-Related Gene Expression in the Hippocampus

**FIGURE 3:**
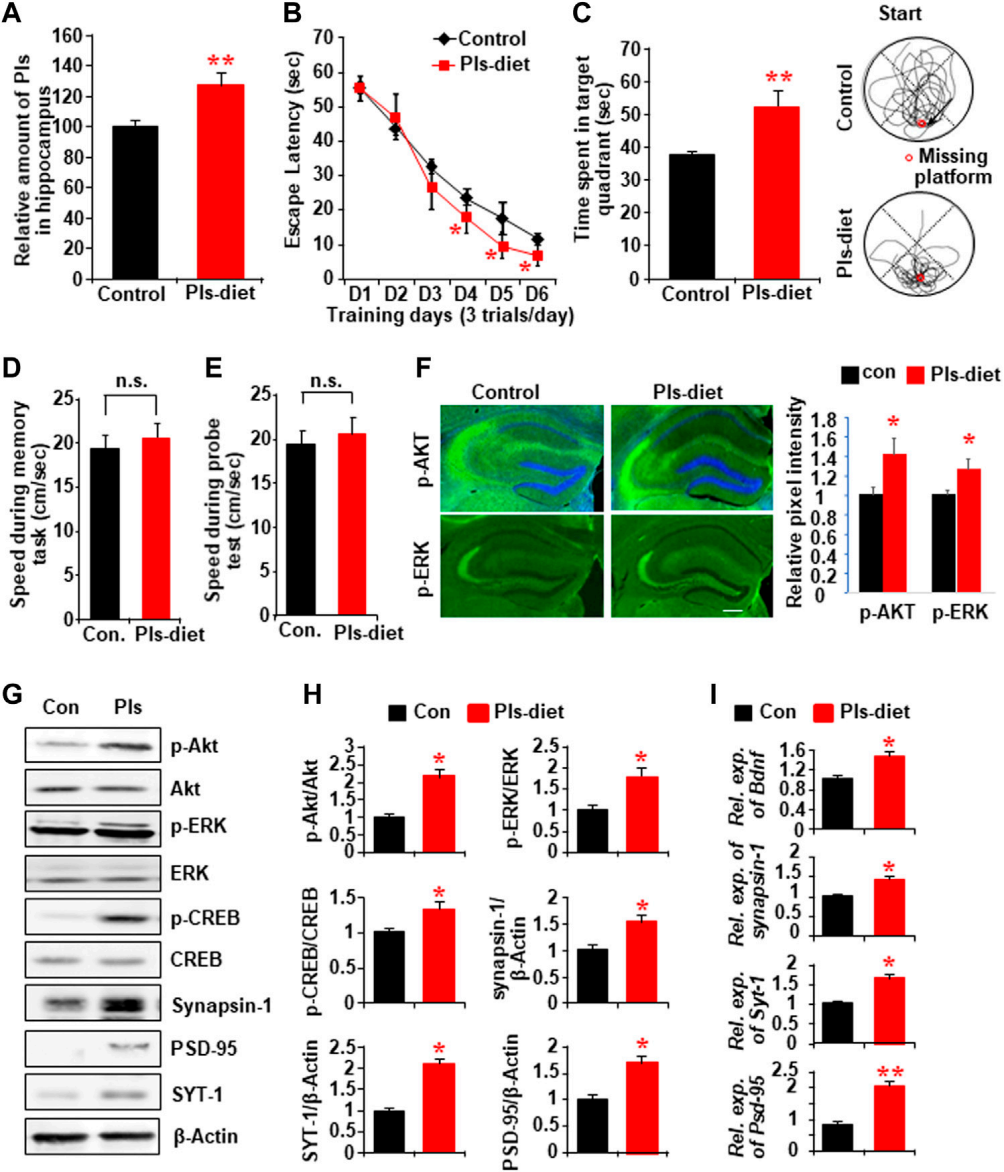
Impact of Pls diet in spatial memory and memory-related protein expression in the hippocampus. **(A)** Mass spectroscopic data show the expression of hippocampal Pls in the mice fed with the diet containing 0.01% Pls for 6 weeks (0.2–0.25 mg Pls/day/mouse, *n* = 5). **(B)** Memory tasks show the change in escape latency by Pls diet. **(C)** The probe test following the memory task shows the changes of time spent in the target quadrant. The right panel shows examples of traces for control (upper) and Pls diet (lower) mice. The red circle indicates the place of the missing platform. **(D,E)** The swimming speed of the mice in both groups during the memory tasks **(D)** and the probe tests **(E)**. **(F)** Immunohistochemistry data show the expression of p-Akt and p-ERK in the control and Pls diet mice hippocampus. Scale bar, 300 μm. The right panel shows the image intensities in relative pixel values. **(G)** Representative Western blotting assays show the protein expression in the control and Pls diet mice hippocampus tissue. **(H)** Quantification data of the panel **(G)** show the relative change in the expression of p-Akt, p-ERK, p-CREB, synapsin-1, PSD-95, and SYT-1 by the Pls diet in the mice hippocampus (*n* = 5). **(I)** Real-time PCR data show the mRNA expression of *Bdnf*, *Synapsin-1*, *Syt-1*, and *Psd-95* in the mice hippocampus (*n* = 5). The data represent mean ± S.E.M. *, *p* < 0.05 and **, *p* < 0.01. The *p* values of panel B were obtained from ANOVA analysis followed by post hoc Bonferroni’s tests. Student’s t-tests were performed in panels **(D,E,F,H,I)**.

After confirming that the reduction of Pls in the hippocampus resulted in memory loss, we investigated whether the oral intake of Pls had any effect on learning and memory. Mice were given 0.01% Pls-containing or control diet for 6 weeks. There were no differences in food intake and body weight increase between Pls-feeding and control mice. After 6 weeks of Pls feeding, we found that Pls content in the hippocampus increased significantly (Figure 3A). The Pls diet improved the learning process in the water maze tasks compared with the control group (Figure 3B). The probe test also showed a significant increase in the exploration time in the target compartment (Figure 3C), suggesting that Pls diet enhanced memory acquisition and maintenance. The enhancement of the memory was not associated with the physical activity of the mice since the swimming speed was not affected by the Pls diet (Figures 3D,E). Consistent with the knockdown data, we observed that Pls diet enhanced phosphorylation of ERK and Akt in the mouse hippocampus, especially in the CA3 region (Figure 3F). Western blotting data showed that the Pls diet increased p-ERK, p-Akt, phosphorylated cAMP-regulated element-binding proteins (p-CREB), synapsin-1, PSD-95, and SYT-1 proteins in the hippocampus (Figures 3G,H). Real-time PCR data also showed the upregulation of mRNA expression of *Bdnf*, *synapsin-1*, *SYT-1,* and *PSD-95* in the hippocampus (Figure 3I). The increases in hippocampal p-Akt and p-CREB expression induced by Pls diet were completely abolished by bilateral microinjection of a phosphoinositide 3-kinase (PI3K)/Akt inhibitor (LY294002) into the hippocampus (0.5 µL of 50 μM solution/site) when measured 24 h after the injection (Supplementary Figure S3A). Quantitative analysis showed that not only p-Akt and p-CREB, but also the Pls-induced increase in *Bdnf* mRNA was suppressed by PI3K/Akt inhibitor (Supplementary Figure S3B). These findings suggest that the Pls diet-induced enhancement of *Bdnf* expression in the hippocampus could be affected by the activation of Akt-induced phosphorylation of CREB proteins.

### Plasmalogens-Feeding Enhances Synaptic Plasticity of Hippocampal Synapses

**FIGURE 4:**
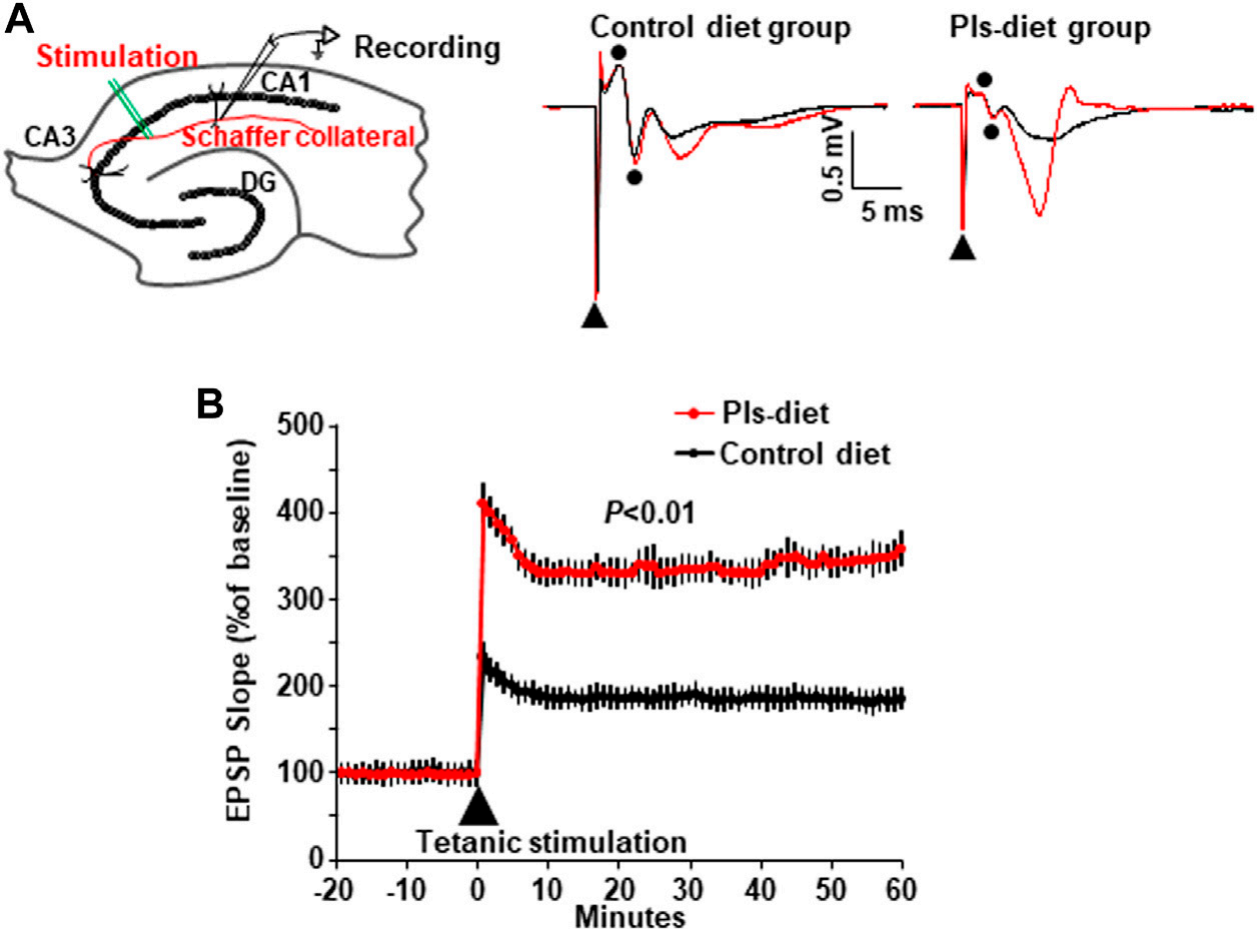
Enhancement of synaptic plasticity by Pls diet. **(A)** The field EPSPs were evoked by stimulation of Schaffer collaterals and recorded in stratum radiatum in the mouse hippocampal slices (left panel). DG, dentate gyrus. The superimposed traces show the EPSP field potentials at time −10 min (black) and 60 min (red) in control and Pls diet mice (middle and right panels). In the traces of control diet [**(A)**, middle], the biphasic wave (dots) just after the stimulus artifact (triangle) was considered to be a fiber volley recorded from nerve fibers since the amplitude was not affected by stimulation (black and red). Then the next downward wave was the first EPSP. The small fiber volley was also observed in the Pls diet. **(B)** Percent changes in the field EPSPs of Pls diet mice (*n* = 15) show a significant enhancement of LTP after the tetanus stimuli compared with the control slices (*n* = 12). The data represent five mice in each group and three to four slices from each mouse were examined). The data represent mean ± S.E.M., *n* = 5 in each group. **, *p* < 0.01 (Student’s t-test).

We investigated hippocampal plasticity by recording long-term potentiation (LTP) of the synaptic transmission. We have recorded the field excitatory postsynaptic potentials (EPSPs) from the stratum radiatum in the area CA1 of hippocampal slices in control and Pls diet mice (Figure 4A, left) and added tetanic stimulation to the Shaffer collaterals to evoke LTP. The basal averages of the first EPSP amplitude and slope of control-diet mice were 0.31 ± 0.02 mV and 0.11 ± 0.01 mV/ms (*n* = 12) and those of Pls diet mice were 0.33 ± 0.02 mV and 0.10 ± 0.01 mV/ms (*n* = 15), respectively. Two superimposed traces showed EPSPs following stimulus artifact (triangle) and fiber volleys (two dots) at time −10 min (black) and 60 min (red) recorded from control and Pls diet mice (Figure 4A, middle and right panels), Percentage changes in field EPSPs showed a significant enhancement of LTP in Pls-feeding mice compared with control-diet mice (Figure 4B). This data suggest that Pls diet could enhance synaptic transmission of the hippocampal neurons.

### Plasmalogens Treatments Enhance the Maturation of Dendritic Spines of Hippocampal Neurons and Enhance Neurogenesis in the Dentate Gyrus

**FIGURE 5:**
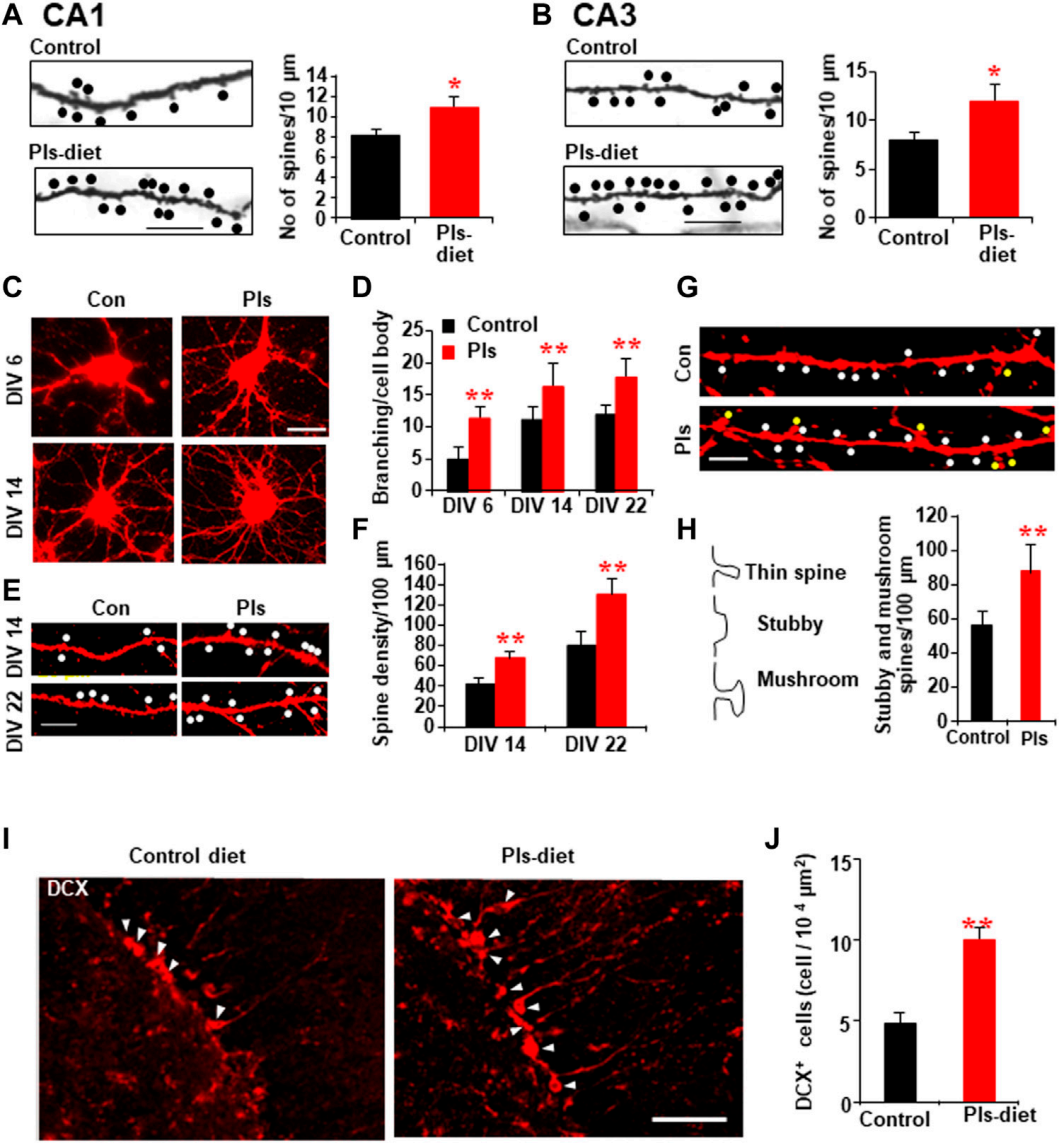
PLs-induced increase in dendritic spines in vivo and in vitro. **(A,B)** Golgi-cox staining shows the representative dendritic-like spines (dots) of CA1 **(A)** and CA3 neurons **(B)** in control and Pls diet mice. Quantification data show the changes in the number of spines in Pls diet mice. Scale bar, 10 μm. **(C–H)** Effects of Pls (5 μg/ml) application on the cultured neuronal cells. Neuronal cells derived from E18 mice embryo were cultured with Pls-containing medium for the indicated days (DIV = disc *in vitro* culture) and stained by Dil to visualize (red color) the morphology of the cells. Pls were added on the DIV3. Branching per cell body **(C,D)** and the number of spines (dots) were enhanced by Pls **(E,F)**. The stubby spines (white dots) and mushroom-shaped spines (yellow dots) were also enhanced by Pls **(G,H)**. Scale bar, 20 μm. **(I)** Immunohistochemistry data show the increased amount of doublecortin (DCX) positive neurons (arrowheads) in the dentate gyrus of Pls diet mice hippocampus. Scale bar, 50 μm. **(J)** Quantification data of panel **(I)** show the significant increase in the DCX-positive neurons in Pls diet mice. The data represent mean ± S.E.M. These values were drawn from the three independent experiments with triplicates. For each sample, more than 20 different locations were photographed by the microscope (40 and 60 X) and scored the spines number per unit length and branching per cell body. *, *p* < 0.05 and **, *p* < 0.01. The *p* values in panels of D and F were calculated by ANOVA followed by Bonferroni’s post hoc tests whereas Student’s t-tests were performed in panels **(A,B,H,J)**.

To examine whether the Pls diet mediated enhancement of synaptic plasticity is correlated with the changes in the synaptic morphology, we performed Golgi-Cox staining and observed a significant increase in the number of dendritic spines (shown as dots) in CA1 and CA3 regions of the hippocampus in the Pls group mice (Figures 5A, B). To see the direct effects, we treated primary hippocampal neurons with Pls. After application of Pls (5 μg/ml) on the DIV (Disc *in vitro*) 3, the number of branching from the neuronal cell body was increased at DIV 6, 14, and 22 (Figures 5C,D), suggesting that Pls enhanced neuronal cell body differentiation to form mature neurons. We further checked the number of dendritic spines in the matured neurons of DIV 14 and DIV 22 since the dendritic spines appeared at these time points. The presence of Pls in the neuronal culture medium increased dendritic spines (dots) significantly at both time points (Figures 5E,F). In addition, the quantity of matured spines called mushroom spines (shown as yellow dots), as well as stubby spines (white dots) at DIV 22, was also found to be increased by the Pls treatments (Figures 5G,H).

Neurogenesis in the hippocampus is known to play a role in memory. To address whether Pls diet enhances neurogenesis, we stained newborn neurons in the hippocampus with the antibody against doublecortin (DCX). There was a significant increase in the DCX-positive neurons in the dentate gyrus region of the hippocampus in the Pls diet mice compared to the control group mice (Figures 5I,J).

To confirm the signaling mechanism of Pls-treatments induced enhancement of dendritic spines, we examined ERK and Akt proteins. Western blotting assays showed significant increases in p-ERK and p-Akt in the neurons of DIV 14 and DIV 22 (Supplementary Figure S4A). The treatments of ERK and Akt inhibitors reduced the Pls-induced increase in dendritic spines at DIV 14 and DIV 22 (Supplementary Figure S4B), suggesting that the Pls could enhance the spine formation by a mechanism dependent on ERK and Akt signaling.

### Plasmalogens Treatments Enhance *Bdnf* Gene Expression by Activating the Transcriptional Factor CREB

**FIGURE 6:**
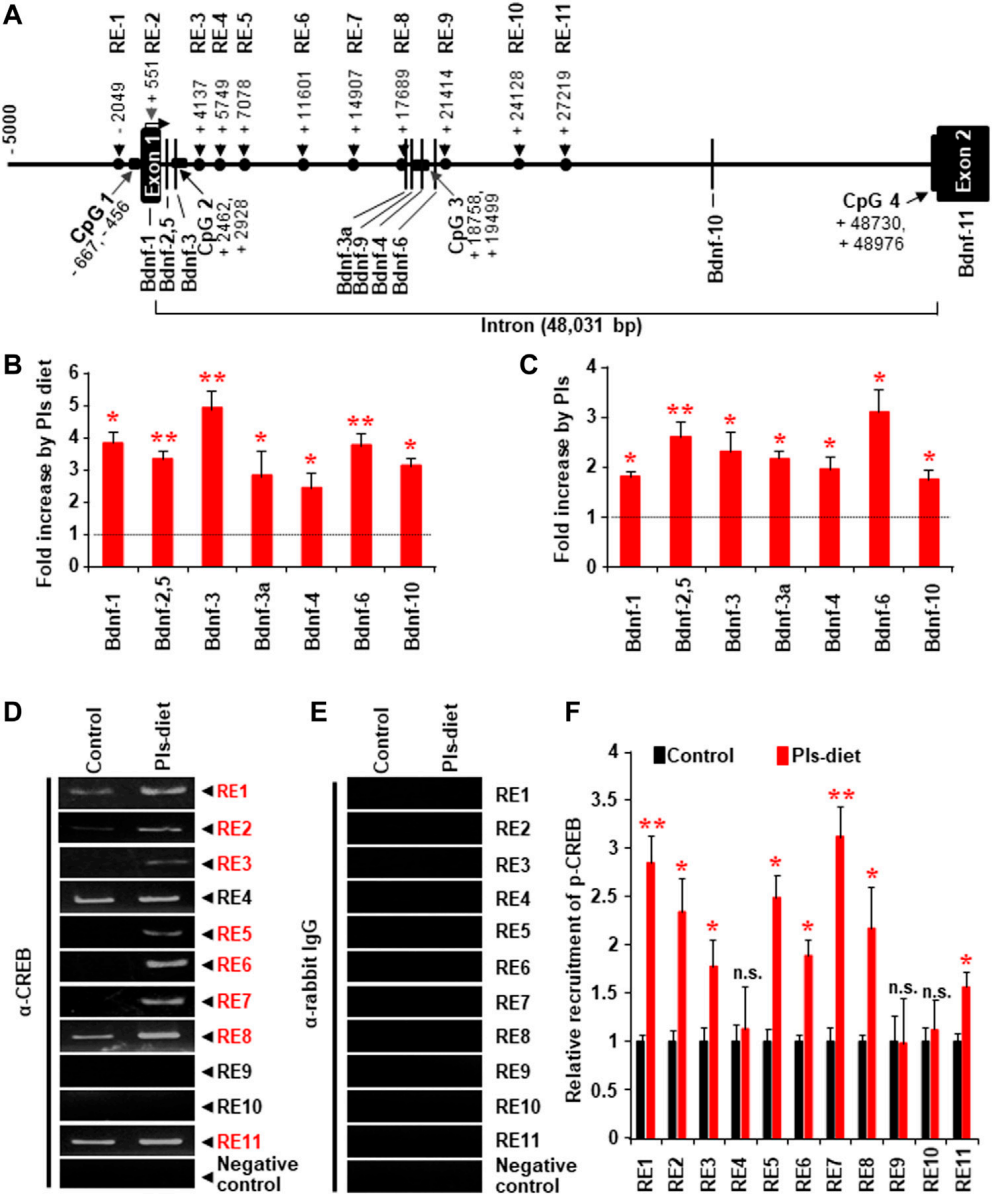
Changes of CREB recruitment onto the Bdnf promoters in the hippocampus tissue by Pls diet. **(A)** The possible CREB recruitment sites (RE indicates responsive element) and the reported promoters onto the mouse *Bdnf* genomic DNA. **(B)** Real-time PCR data show the relative increase in the *Bdnf* transcripts from the different promoters by Pls diet in the mice hippocampus. The values show relative increases compared with the control diet (*n* = 5, in each group). **(C)** Real-time PCR data show the relative increase in *Bdnf* transcripts in primary neuronal cells (at DIV 22) cultured with Pls (5 μg/ml). Pls were added on the DIV3. **(D,E)** ChIP assays show the relative pulldown of the genomic DNA fragments by the phosphorylated CREB antibody **(D)** and the control rabbit IgG **(E)** in the hippocampus of a control diet and Pls diet mice. **(F)** Real-time PCR data following the ChIP assays show the relative recruitment of the p-CREB onto the different responsive elements. In panels, **(B,C,F)** the data represent mean ± S.E.M. (*n* = 5, *, *p* < 0.05 and **, *p* < 0.01). The *p* values were calculated by ANOVA followed by Bonferroni’s post hoc tests to compare the multiple groups.

We investigated whether Pls-treatments could induce *Bdnf* gene expression by promoting the recruitment of CREB proteins onto the *Bdnf* promoter regions. Genomic sequence analysis revealed that there are eleven possible CREB recruitment sites onto the mouse *Bdnf* genomic regions (Figure 6A). We found that Pls diet enhanced expression of all the *Bdnf* isoforms in the mouse hippocampus (Figure 6B) and in the primary cultured neurons at DIV 22 treated with Pls (Figure 10C), suggesting a possibility that Pls could enhance CREB recruitments onto those promoter sites. Extensive ChIP assays data showed that Pls diet significantly enhanced CREB recruitments onto the putative binding sites of RE (Responsive elements) 1, 2, 3, 5, 6, 7, 8, and 11 (Figures 6D–F). Negative control was performed to amplify the genomic region between the exon10 and 11, which did not show any recruitment of CREB (data not shown). IgG control of the ChIP assays did not show any nonspecific recruitments (Figure 6E) onto these recruitment sites. To our knowledge, it is the first report of comprehensive ChIP data that suggests a CREB-mediated regulation of the *Bdnf* isoforms in neuronal cells.

### Plasmalogens Are Enriched in the Lipid Rafts and Enhance TrkB Expression in the Rafts to Accelerate BDNF Signaling

**FIGURE 7:**
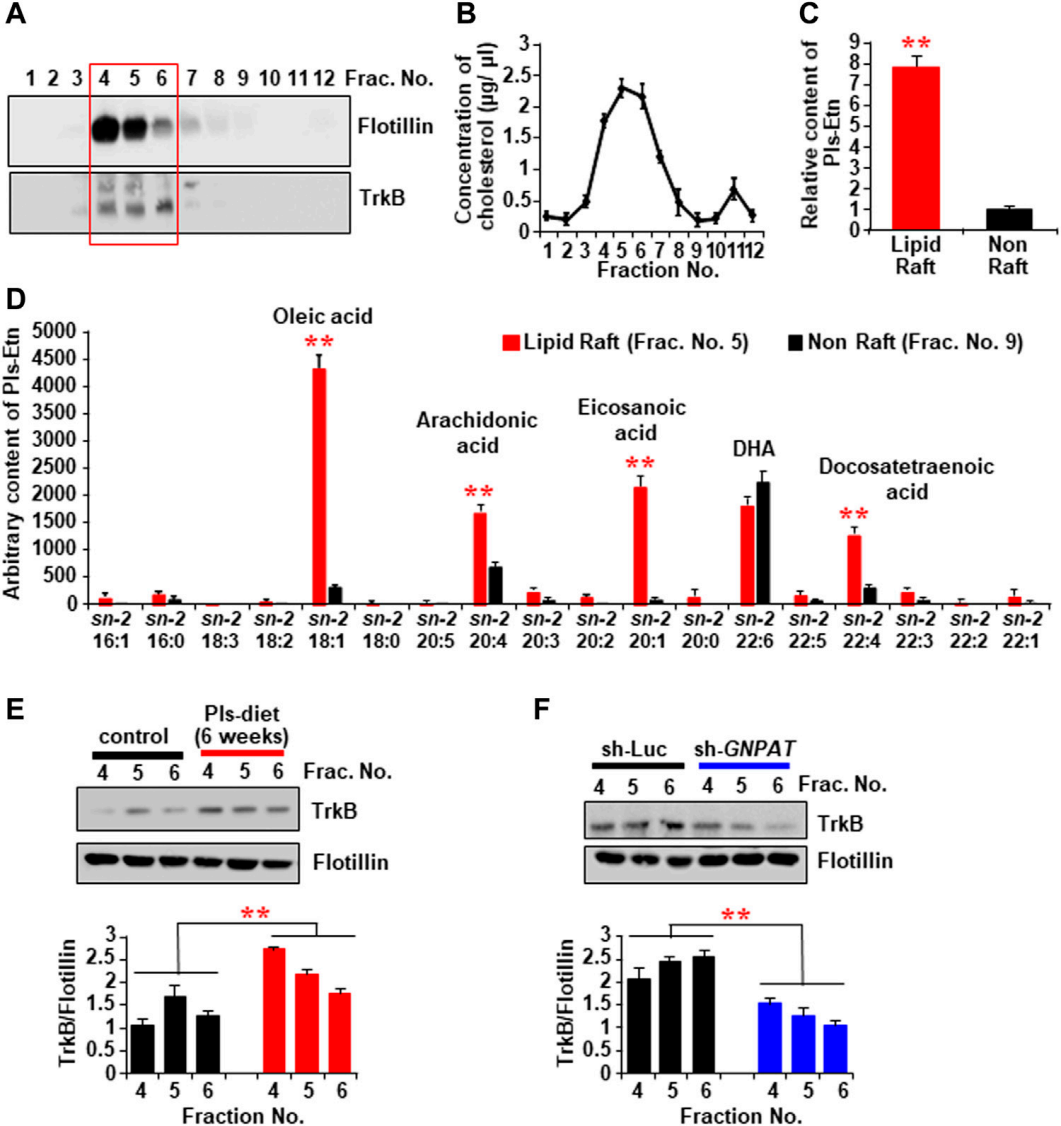
The changes of TrkB recruitment into the lipid rafts of hippocampal tissues by the Pls diet. **(A)** Western blotting data show the expression of TrkB protein in the lipid raft fractions (sucrose gradient fraction no. 4, 5, and 6) of the hippocampus tissue. The Flotillin was used as a lipid raft marker. **(B)** Concentration of cholesterol in the sucrose gradient fractions of the hippocampus tissue. **(C)** LC-MS analysis shows the total Pls content in the rafts and non-raft factions of the hippocampus tissue. **(D)** LC-MS analysis shows the distribution of *sn-2* fatty acid components of Pls in the raft and non-raft fractions. **(E)** Western blotting data show the expression of TrkB in the lipid raft fractions in the control and Pls diet mice hippocampus. **(F)** TrkB contents in the hippocampal lipid raft fractions of control and sh-*GNPAT* group. The data represent mean ± S.E.M. The values were drawn from the three independent experiments with triplicates (*n* = 3). *, *p* < 0.05 and **, *p* < 0.01. The *p* values in the panels **(C,D)** were calculated by Student’s t-test whereas ANOVA test (followed by Bonferroni’s post hoc tests) was performed to get the significance values in the panels **(E,F)**.

It has been known that the receptor for BDNF, TrkB, can be recruited in lipid raft microdomains of the cell membrane to induce BDNF signaling (Suzuki et al., 2004; Assaife-Lopes et al., 2010). The membrane fraction assays of the mouse hippocampal tissues showed a significant amount of TrkB in the lighter upper fractions (Fraction No. 4, 5, and 6), which were enriched in lipid raft marker protein Flotillin (Figure 7A) as well as cholesterol (Figure 7B). The LC-MS assays revealed that the lipid raft fractions have more Pls than the non-raft fractions (fraction no. 8, 9, and 10) (Figure 7C). We, therefore, suggest that a high content of Pls-Etn in the hippocampal lipid rafts might promote BDNF-TrKB signaling from the lipid rafts. The LC-MS assay also showed that Pls containing monounsaturated fatty acids such as oleic acid and eicosanoic acid, and ω-6 polyunsaturated fatty acids (PUFA) such as arachidonic acid and docosatetraenoic acid were enriched in lipid rafts compared with non-rafts whereas the amount of ω-3 PUFA, docosahexaenoic acid-containing Pls was similar in both the rafts and non-rafts (Figure 7D).

We examined the TrkB expression in the lipid rafts of hippocampal tissues in Pls diet mice and found an increase in TrkB proteins in the raft fractions compared with the control-diet mice (Figure 7E). We also detected a reduction of TrkB protein in the hippocampus raft fraction in *GNPAT* knockdown mice (Figure 7F), suggesting that hippocampal Pls could control TrkB expression level in the lipid raft fractions. These cumulative data, together with the evidence that expression of BDNF itself is dependent on Akt/ERK activation (Supplementary Figure S2), suggest that Pls may not only induce BDNF expression through the Akt/ERK activation but also enhance the BDNF actions through the TrkB expression in the lipid rafts.

### Plasmalogens-Induced Enhancements of Learning and Memory Are Dependent on the BDNF-TrkB Signaling Pathway

**FIGURE 8:**
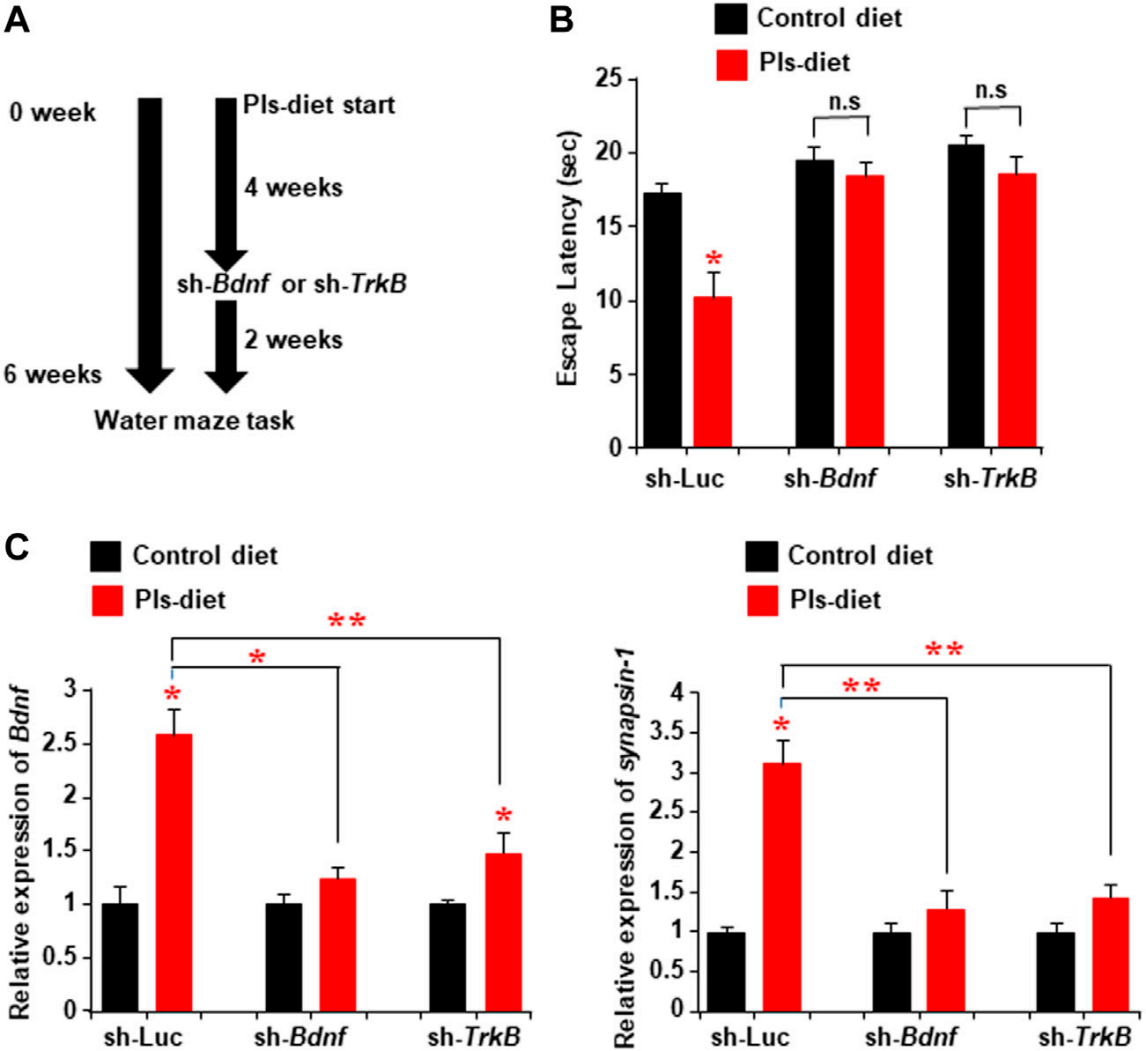
Attenuation of Pls-mediated memory enhancement by knockdown of hippocampal *TrkB* or *Bdnf* expression. **(A)** Mice were subjected to the Pls feeding for 4 weeks followed by the sh-*Bdnf* or sh-*TrkB* injection bilaterally into the hippocampus. The water maze task was performed 6 weeks after Pls feeding. **(B)** Water maze data show the difference in escape latency between the experimental group mice. The data represent the mean values of the swimming time to reach the goal on the fourth-day trails (3 trails per day). **(C)** Real-time PCR data show the expression of *Bdnf* and *synapsin-1* in the hippocampus tissues of the mice. The data represent the mean ± S.E.M. Each experiment included more than five mice. *, *p* < 0.05 and **, *p* < 0.01 (ANOVA followed by Bonferroni’s post hoc tests).

To address whether the Pls-induced enhancements of learning and memory are dependent on BDNF-TrkB signaling in the hippocampus, we injected the lentiviral sh-RNA vectors against either *TrkB* or *Bdnf* bilaterally into the hippocampus 4 weeks after the start of Pls-feeding in mice. The Pls diet was continued for additional 2 weeks following the injection. The water maze test was performed after a total of 6 weeks of Pls diet (Figure 8A). Pls diet-mediated improved learning was attenuated in the mice injected with sh-Bdnf and sh-TrkB (Figure 8B). In addition, the Pls-induced enhancement of *Bdnf* and *synapsin-1* expression was canceled in the *Bdnf* and *TrkB* knockdown mice (Figure 8C). These findings suggest that the Pls-induced enhancements of learning and memory are dependent on BDNF-TrkB signaling in the hippocampus.

### Effects of the Plasmalogens Derived From Scallop in Learning and Memory

**FIGURE 9:**
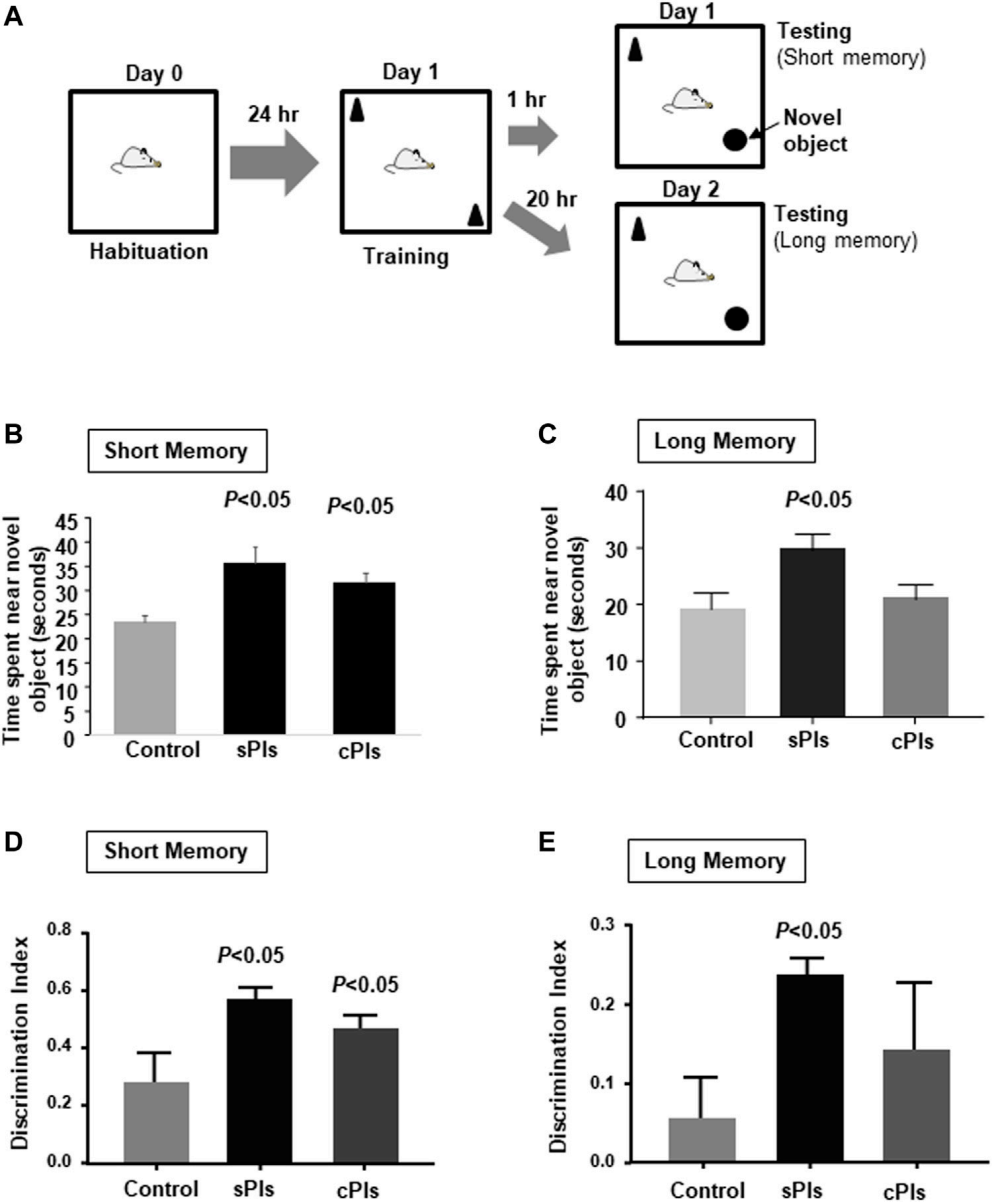
Memory enhancing effects of plasmalogens derived from scallop and chicken. **(A)** Diagram of the novel object recognition test. The mice were subjected to the cPls (Pls from chicken) and sPls (Pls from scallop) drinking (0.01% w/v) for 6 weeks before the tests. **(B)** The data show the time spent near the novel platform when the mice were placed in the chamber after 1 h of the testing phase. **(C)** The data show the average time spent near the novel platform when mice were subjected to the testing phase of 18 h following the training phase. **(D)** The data show the discrimination index in the short-term memory. **(E)** Discrimination index of the long-term memory. Each experimental group contained five mice (*n* = 5). The *p* values were calculated by ANOVA followed by Bonferroni’s post hoc tests to compare the multiple groups.

Recent clinical studies showed that scallop-derived Pls (sPls) improved cognition among AD patients (Fujino et al., 2017; Fujino et al., 2018). To screen the effects of sPls, we have performed the learning and memory tests and compared with the effects of the Pls derived from chicken (cPls). We employed a novel object recognition test to check short and long memory (Figure 9A). Both the Pls treatments (sPls and cPls) improved the novel object exploration time in the short-term memory training (Figure 9B). sPls-treated mice showed an increase of novel object exploration time during the long-term memory training, whereas cPls-treated mice failed to do so (Figure 9C). When we examined the discrimination index (DI), we observed the same effect in short-term memory training (Figure 9D). In the long-term memory training, the sPls-treated mice showed a significant increase in DI values compared to the control group (Figure 9E). These data suggest that scallop-derived Pls which are rich at DHA Pls showed relatively better effects than chicken-derived Pls in the long-term memory performance.

## Discussion

The mechanism of progressive memory loss in patients with AD remained largely unknown. It has been shown that patients with AD have reduced Pls-Etn levels in the cortex and hippocampus (Ginsberg et al., 1995; Guan et al., 1999; Han et al., 2001), suggesting a possibility that Pls content in the hippocampus might regulate the memory process in humans. Interestingly, oral intake of Pls has improved cognition among AD patients (Fujino et al., 2017; Fujino et al., 2020). This evidence indicated a possibility that Pls could play a significant role in the memory process of the human brain, especially in the hippocampus. Our recent findings of the mice study clearly showed that the reduction of hippocampal Pls reduced learning and memory associated with the reduction of BDNF-TrkB signaling. To our knowledge, it is the first report indicating the possibility that a reduction of brain Pls could be a risk factor for memory loss in humans. The patients with AD also showed a reduction of BDNF expression in the brain (Peng et al., 2009). Since BDNF plays a crucial role in synaptic plasticity and neuronal survival (Peng et al., 2009; Cunha et al., 2010; Lu et al., 2014), we propose that brain Pls can improve hippocampus-dependent learning and memory by regulating the BDNF expression. As with the neuroprotective effects of BDNF, we previously found that Pls inhibited apoptosis of neuronal cells (Hossain et al., 2013). It has been known that BDNF treatments could induce synaptic plasticity as measured by the increase of LTP (Miao et al., 2021). Therefore, we suggest that Pls treatments-mediated increase in BDNF expression in the hippocampus could enhance the synaptic transmission of hippocampal neurons as measured by LTP assays. It was known that BDNF treatments could enhance spine maturation of hippocampal neurons, which is similar to the effects of the Pls treatments, suggesting further that the Pls treatments showed BDNF-like effects. We also noticed that Pls diet-induced learning and memory was inhibited when hippocampal *Bdnf and TrkB* were knockdown, suggesting that Pls could enhance memory by increasing the BDNF signaling n the hippocampus.

The precise mechanism of Pls diet-induced *Bdnf* expression in the hippocampus remained unknown. We observed a slight increase of hippocampal Pls by the Pls diet, suggesting that this increase of Pls content could enhance the *Bdnf* expression in the hippocampus. Activation of Akt is known to be an upstream event of CREB phosphorylation and the transcriptional regulation of *Bdnf* gene (Du and Montminy, 1998). We previously reported that Pls enhanced phosphorylation of Akt and ERK protein in neuronal cells (Hossain et al., 2013). Inconsistent with the previous findings, the Pls treatments have been shown to enhance the expression of p-ERK and p-Akt protein in the hippocampus tissues. In addition, the Inhibition of Akt and ERK attenuated the Pls-induced *Bdnf* expression and reduced the dendritic spine formation, suggesting that Pls-mediated activation of ERK and Akt proteins could play a significant role in the memory process possibly by inducing *Bdnf* expression. In the present study, we cannot claim that the Pls treatment-induced alteration of cellular signaling is a direct effect of Pls because Pls can be degraded in the cells to produce PUFA and lyso-Pls. Further studies will be necessary to address whether the PUFA and lyso-Pls can regulate the cellular signaling in our experimental condition. However, we could suggest that Pls might function as ligands to activate the cellular signaling. It can be supported by the previous findings that membrane-bound GPCRs were critical to induce Pls-mediated cellular signaling in the neuronal cells (Hossain et al., 2016). The previous report showed that CREB was localized at upstream of exon-1 of *Bdnf* gene (Zheng et al., 2012). However, we detected the CREB recruitment in other regions within the *Bdnf* genomic region and found the transcriptional upregulation of various *Bdnf* isoforms. These findings suggested that Pls treatments could induce the expression of the *Bdnf* isoforms by promoting the CREB recruitments onto those promoter regions of *Bdnf*. It has been known that the Bdnf transcription in neuronal cells can be regulated by other transcriptional factors including AP-1 (Tuvikene et al., 2016), In addition to the CREB recruitment, the promoter activity can also be regulated by the epigenetic factor such as histone acetyl transferase (HAT) and it has been known that the *Bdnf* expression was critically controlled by the treatments of HAT inhibitor (SAHA) (Mattson and Meffert, 2006; Guan et al., 2009; Sartor et al., 2019). We have not studied whether Pls treatments could regulate AP-1 and HAT activity and future experiments will be necessary to address this issue. The involvement of *Bdnf* isoform in the learning and memory process is complex and further experiments will be necessary to identify which *Bdnf* isoform is involved in the regulation of memory by the Pls treatments.

It has been shown that Pls are important for the organization of cholesterol-rich lipid raft microdomains in the membrane (Thai et al., 2001; Pike et al., 2002), which is known to participate in cellular signaling (Allen et al., 2007). Pls-deficient mice lacking *GNPAT* showed disruption of these membrane microdomains (Rodemer et al., 2003). Our data showed the localization of Pls in the lipid rafts of mice hippocampus tissue where TrkB was found to be accumulated. Our results, increased TrkB localization by the Pls-diet, could suggest that an association of Pls to the raft could enhance the localization of TrkB because we detected a reduction of TrkB in the Gnpat knockdown hippocampus. The localization of membrane proteins including TrkB in the lipid-raft microdomains is known to enhance Akt and ERK signaling (Suzuki et al., 2004; Cao et al., 2013; Assaife-Lopes et al., 2014). Therefore, it is likely that the Pls could facilitate TrkB-signaling in the lipid rafts to induce *Bdnf* expression. However, the mechanism of how the Pls enhanced TrkB localization in the lipid-raft remained unknown. We have observed that the knockdown of TrkB and Bdnf in the hippocampus resulted in an increase in escape latency, suggesting that the TrkB signaling can regulate the memory which can be supported by the published literature (Yamada and Nabeshima, 2003; Wang et al., 2019).

In the present study, we observed that scallop-derived Pls treatments significantly enhanced the short-term and long-term memory whereas chicken-derived Pls only enhanced the short-term memory. We still do not know why the cPls treatments in contrast to the sPls treatments failed to show a significant effect at long-term memory. It is known that sPls are enriched in DHA-containing Pls which are absent in cPls (Youssef et al., 2019). We previously found that DHA-containing Pls were more effective in inhibiting the microglial activation (Ali et al., 2019; Youssef et al., 2019). The neuroinflammation, characterized by microglial activation in the brain, is a well-known pathological event associated with memory impairment in patients with AD (Heneka et al., 2015). Therefore, we suggest that the presence of DHA-containing Pls in the sPls could have a better effect in reducing neuroinflammation in the brain and improving the long-term memory than the cPls. We still do not know whether the DHA-Pls content in the lipid-raft could be involved in the long-term memory as we also observed enrichment in the non-raft fraction. Further experiments will be necessary to address whether the DHA-Pls content in the sPls is important for long-term memory. Our previous study showed that the reduction of the Pls induced neuroinflammation in the hippocampus (Hossain et al., 2017), suggesting that the deficiency of memory by the Pls knockdown in hippocampus might be due to the neuroinflammation. Although further studies will be necessary to address the mechanism of memory-enhancing effects by sPls and cPls, our findings indicate that there might be a difference in pharmacological function of Pls depending on the source. Though the Pls treatments enhanced cellular signaling to induce *Bdnf* expression and neuronal differentiation (*in vitro* studies) which might explain that a reduction of brain Pls could lead to the loss of memory, our present findings have the limitation in understanding how the oral intake of Pls improved memory (*in vivo* effects) because it remains unknown whether Pls can cross the blood-brain barrier (BBB). It has been known that PUFA can improve cognitive function (Yurko-Mauro et al., 2015; Pifferi et al., 2021), The sn-2 position of Pls can be cleaved by enzymatic reaction in the blood giving rise to PUFA, suggesting a possibility that PUFA derived from the dietary Pls could also improve learning and memory. Although PUFA can cross the BBB, it has been known that PUFA can also alter the intestinal microbial flora and gut hormone secretion including glucagon-like peptide-1 (GLP-1) (Shida et al., 2013; Menni et al., 2017; Parolini, 2019), which could stimulate the vagus nerve to modulate learning and memory (Clark et al., 1999; Hossain et al., 2020). Therefore, PUFA and the dietary Pls might have a significant effect to alter the gut microbial flora and secretion of gut hormones to regulate learning and memory. Further studies will be necessary to address whether the dietary Pls could affect the gut-brain axis to improve learning and memory in mice. However, our present results could suggest that a reduction of brain Pls, which is common in patients with AD, might lead to a reduction in learning and memory.

Our cumulative pieces of evidence suggest that a reduction of hippocampal Pls could be a risk factor for AD. These lines of evidence of Pls-mediated memory improvement in experimental mice and patients with AD (Fujino et al., 2017) suggest that the oral intake of Pls could be a potent therapeutic strategy to improve memory by enhancing the synaptic plasticity in the hippocampus.

## Materials and Methods

### Animal, and Cell Treating Reagents

All the animal experiments were followed by the guidelines provided by the Committee on the Ethics of Animal Experiments, Kyushu University, and carried out by the Guidelines provided by the National Institute of Health in the United States regarding the care and use of animals for experimental procedures. All efforts were made to minimize animals’ suffering and the number of animals used for the study.

Male C57BL/6 mice (8 weeks old) were used for *in vivo* study. Primary hippocampal neurons were prepared from the E-18 embryo of mice. After dissection of anesthetized pregnant mice, the meninges of the embryo were removed carefully. The hippocampus was cleared with the surrounding cortex and dissolved in trypsin solution containing phosphate-buffered saline (PBS), bovine serum albumin (BSA), and glucose at 37°C for 15 min. FBS was used to neutralize the trypsin activity. The hippocampus was then dissociated in neurobasal medium (GIBCO) supplemented with B27 (GIBCO) by appropriate pipetting using different pour-sized Pasteur pipettes. The dissociated neurons were then cultured on poly-D-lysine coated glass coverslips (30,000 cells/15 mm coverslip) with the neurobasal medium in a 5% CO_2_ humidified incubator. On DIV (Disk *In Vitro*) 3, 90% of the cultured medium was replaced with a B27 free neurobasal medium. Cytosine arabinoside (Ara-C) purchased from Sigma was added to DIV three primary neurons at a concentration of 1 μM to inhibit microglial proliferation. More than 95% pure primary hippocampal pyramidal neuronal cells (on DIV 21) were used as primary neurons (Hossain et al., 2013). To visualize the neuronal cells, we performed Dil (tetramethylindocarbocyanine perchlorate) staining by following the recommended protocol (Invitrogen). PI3K/AKT and MAPK ERK Kinase (MEK) inhibitors named LY294002 (Catalog number #9901) and U0126 (#9903), respectively, were purchased from Cell Signaling Technology (Boston, MA, United States) and human recombinant BDNF from WAKO (Osaka, Japan).

### Preparation of Plasmalogens Containing Food and Water

The Pls used in the present study were extracted from chicken and scallop by using a previously reported method (Mawatari et al., 2009) and kindly donated by Central Research Institute, Marudai Food Co. Ltd. (Osaka, Japan) and B&S Corporation Co., Ltd. (Tokyo, Japan). The purified Pls consisted of 96.5% Pls-Etn, 2.5% Pls-Cho, 0.5% sphingomyelin (SM) and 0.5% other phospholipids (Mawatari et al., 2007). The composition of fatty acids of Pls-Etn was analyzed using the high-performance liquid chromatography method (Mawatari et al., 2009) and shown in Tables 1, 2. Pls (0.01%)-containing pellet food was prepared by Clea Japan Inc. (Tokyo, Japan). The composition of control and chicken Pls containing food was shown in Table 2. We have used Pls (chicken and scallop derived Pls) dissolved (0.01% w/v) in drinking water for the novel object recognition test.

**TABLE 1:** The fatty acid composition of the Pls-Etn in the purified Pls from chicken (cPls) and scallop (sPls).

Fatty acid	sPIs (% w/w)	cPIs (% w/w)
Linoleic acid	0.1	5.1
Oleic acid	2.5	33.1
Eicosapentaenoic acid, EPA	27.8	0.9
Arachidonic acid	24.9	21.9
Docosahexaenoic acid, DHA	37.1	23.8
Other acids	7.6	15.2
Total	100.0	100.0

**TABLE 2:** Composition of control and chicken derived Pls (0.01% w/w) containing pellet food.

Component	Control food (%) (AIN-93M, Clea, Japan)	PIs—Containing food (%)
cornstrach	61.1	60.6
Milk casein	14.0	14.0
Sugar	10.0	10.0
cellulose	5.0	5.0
Soybean oil	4.0	4.5 (0.01% PIs)
Mineral mixture	3.5	3.5
Vitamin mixture	1.0	1.0
α-cornstarch	1.0	1.0
Choline bitartrate	0.22	0.22
L-cystine	0.18	0.18
Total	100.0	100.0

### Preparation of sh-RNA Lentiviruses and *in vivo* Injection Into Mouse Brain

The sh-RNA sequences were cloned into the pLL3.7 lentiviral vector following the protocol provided in the Addgene website (Plasmid No 11795 (Rubinson et al., 2003)). The target sequences were as follows: sh-*GNPAT* (5′-GTC​CCA​ATT​AGC​ATC​AGT-3′), sh-*Bdnf* (5′-AGT​CCC​GGT​ATC​CAA​AGG-3′), sh-*TrkB* (5′-CCT​GTA​CAT​GAT​GCT​CTC-3′) and sh-Luc (5′-CTT​ACG​CTG​AGT​ACT​TCG​AG-3′). For the viral constructs, we transfected the Hek-293T cells with the cloned pLL3.7 vectors along with the vectors pMD2. G (Addgene plasmid 12,259), pRSV-Rev (Addgene plasmid 12,253), and pMDLg/pRRE (Addgene plasmid 12,251). The cells supernatant was centrifuged at 24,000 rpm for 3 h at 4°C and the viral pellet was dissolved in PBS (1% BSA). After checking the viral titer in N2A cells, 5 × 10^5^ TDU (transduction units) were injected stereotaxically into the bilateral hippocampus through the 30-gauge stainless steel tube at an injection rate of 0.05 μL/min after making holes on the temporal bone. The stereotaxic coordinates of the hippocampus were A, 1.67 mm posterior to bregma; L, 1.25; and H, 2.0 from the surface of the brain. The wound was sutured and treated with an antibiotic. After the experiments, the injection site was confirmed by immunohistochemistry.

### Morris Water Maze Task

Morris water maze task was performed in a black plastic circular pool (diameter, 100 cm; wall height, 45 cm) containing water at 22–23°C. The pool was surrounded by a gray curtain wall, on which 3 rectangular, triangle, and circular drawings brightly illuminated were placed and served as the spatial cues. A circular, transparent plastic platform (diameter, 12 cm) was placed in one quadrant of the pool 2 cm below the surface of the water. After the pre-swimming task performed 1 day before the maze task, mice were released from one of four randomly chosen starting points in the circular pool to search for the hidden escape platform for 60 s. They were allowed to rest for 30 s on the platform after they found it. If mice could not find the platform within 60 s, the experimenter placed the animal on the platform for 10 s. Mice were then placed for 30 min in a waiting cage for the next trial and were dried under a heating lamp. The mice received 4 trials in a day for 4 consecutive days. There were no mice that did not show motivation for swimming (floating behavior). The mice were tracked by an infrared-sensitive camera connected to a maze analysis unit (SMART, Panlab S. L., Barcelona, Spain). A probe test was performed after the end of the maze task, in which the mice were allowed to search for 60 s in the absence of the platform. The duration of cumulative time they spent in the target quadrants was measured in the probe test. In the experiments of Pls diet mice, they received three trials in a day until day 6 (D6).

### Novel Object Recognition Test

The novel object recognition test was performed according to the published paper (Lueptow, 2017). Mice were subjected to Pls drinking (cPls and sPls) at the dose of 0.01% w/v for 6 weeks. Before starting the habituation phase, mice were kept in the experimental room for 1 hour to adjust the room condition. For the habituation phase, mice were placed in a chamber (65 cm × 65 cm x 30 cm) and allowed to explore for 10 min. For the training phase, mice were placed in the same chamber containing two similar objects for 5 min while being recorded by camera overhead. To remove the odor left by the previous mice, we cleaned the chambers with 70% alcohol. For the testing of short-term memory, mice were placed in the same box with the novel object for 5 min after 1 hour of the training phase. To check the long-term memory, mice were placed in the chambers after about 18 h of the training phase. The discrimination index was calculated by the formula: (time exploring near the novel object—time exploring the familiar object)/(time exploring the novel object + time exploring the familiar object).

### Real-Time PCR Analyses

Mice were deeply anesthetized with pentobarbital (50 mg/kg) and transcardially perfused with sterile PBS. For each animal, the brain was removed and the hippocampus was dissected in a dish filled with ice-cold PBS. The tissue samples were stored at −80°C until PCR analyses. Total RNAs were extracted from tissues using TRIZOL reagents (Life Technologies) following standard protocols. cDNAs were then prepared from the purified total RNAs using ReverTra Ace qPCR RT Kit (Toyobo). All the Real-time qPCR reactions were carried out by SYBR Premix ExTaq (RR420Q, Takara) followed by the real-time quantification using 7,500 Real-Time PCR System (Applied Biosystems). The specific primer sets used for amplifying each mouse gene were as follows: *GNPAT*, forward 5′-GCG​CTG​TCT​CAG​ACT​TCC​AT-3′ and reverse 5′-GGA​GGA​CAT​CCA​CAC​CTG​TC-3′; *Bdnf*, forward 5′-TGC​AGG​GGC​ATA​GAC​AAA​AGG-3′ and reverse 5′-CTT​ATG​AAT​CGC​CAG​CCA​ATT​CTC-3′; *synapsin-1*, forward 5′- GGC​CTA​CAT​GAG​GAC​ATC​AG-3′ and reverse 5′-ACC​ACA​AGT​TCC​ACG​ATG​AG-3′; *synapsin-2* forward 5′-CAG​GTA​CTT​CGG​AAT​GGC​AC-3′ and reverse 5′-CAA​ATG​CAT​GCT​GTC​GGA​T-3′; *synaptotagmin-1 (SYT-1)*, forward 5′-CAC​CGT​GGG​CCT​TAA​TTG​C-3′ and reverse 5′-TGT​TAA​TGG​CGT​TCT​TCC​CTC-3′; *Ca* ^*2+*^ */calmodulin-dependent protein kinase II-α* (*CamKII-α*), forward 5′- ATG​CTC​CGT​CCA​AAT​ACC​CTC​C-3′ and reverse 5′-GCA​GTG​GTC​ATT​CAA​GTT​CAC​AGC-3′; *Homer-1*, forward 5′-CTA​TAT​TCT​CCG​CGC​AAC​CT-3′ and reverse 5′-GCA​ACT​CAA​CAA​GGC​AGA​CA-3′; *PSD-95*, forward 5′-CAC​CGA​CTA​CCC​CAC​AGC-3′ and reverse 5′- ACT​GGC​ATT​GCG​GAG​GTC-3΄; and *β-Actin*, forward 5′-CAC​TGT​GCC​CAT​CTA​CGA-3′ and reverse 5′-CAG​GAT​TCC​ATA​CCC​AAG-3′. To amplify the different isoforms of mouse *Bdnf* genes, we have used the following primers *Bdnf1*, forward 5′-CTG​TAG​TCG​CCA​AGG​TGG​AT-3′ and reverse 5′-AGA​AGT​TCG​GCT​TTG​CTC​AG-3΄; *Bdnf2/5*, forward 5′-TGG​AAG​AAA​CCG​TCT​AGA​GCA-3′ and reverse 5′-TCT​GTC​CAA​GGT​GCT​GAA​TG-3΄; *Bdnf3*, forward 5′-CGA​TCC​TCG​ATG​GAT​AGT​TCT​T-3′ and reverse 5′-CTT​CCC​TTG​AGA​AGC​AGG​AG-3΄; *Bdnf4*, forward 5′-CTG​GGA​GGC​TTT​GAT​GAG​AC-3′ and reverse 5′-CAT​TGT​TGT​CAC​GCT​TCT​GG-3′; *Bdnf6*, forward 5′-CTC​CAG​GAC​AGC​CTG​TAT​CC-3′ and reverse 5′-TCC​CGG​ATG​AAA​GTC​AAA​AC-3′; and *Bdnf10*, forward 5′-GGA​ACC​ACC​AGT​TTT​CTC​CA-3′ and reverse 5′-TGT​GTG​GGT​AGA​TGC​CAA​AA-3′. The mRNA expression was calculated using delta-delta Ct method and the expressional level of mRNA was normalized with the endogenous expression of mouse *Gapdh* (forward: 5′-CAA​TGT​GTC​CGT​CGT​GGA​TCT-3′ and reverse: 5′-GTC​CTC​AGT​GTA​GCC​CAA​GAT​G-3′).

### Western Blotting Assays

Western blotting assays were carried out by standard protocols (Hossain et al., 2013) using antibodies against GNPAT (ab75060, Abcam, Cambridge, MA), CREB (9,197, Cell signaling), p-CREB (9,191, cell signaling), Akt (610,860, BD Biosciences), p-Akt (s-473, 4,060, Cell signaling), ERK (4,695, Cell signaling), p-ERK (4,370, Cell signaling), pro-BDNF (ANT-006, alomone labs), Synapsin-1 (AB1543, Millipore), SYT-1 (AB5600, Millipore), PSD-95 (MAB1598, Millipore), Flotillin (610,820, BD Biosciences), TrkB (610,102, BD Biosciences) and β-Actin (sc-47778, Cell Signaling).

Hippocampal tissues were dissected in the same way as PCR analyses and lysed using the RIPA buffer (1% NP40, 0.5% sodium deoxycholate, and 0.1% SDS dissolved in 1X TBS) mixed with the complete protease inhibitor cocktail tablets (Roche). After 30 min on ice with the lysis buffer, cells were subjected to brief sonication at 4°C followed by centrifugation at 15,000 rpm for 10 min to remove insoluble cell debris. For subcellular fractionation assays, cells were suspended in mild buffer (0.5% NP40, 1 mM EDTA, and 10 mM Tris-HCl) followed by centrifugation at 15,000 rpm and the supernatant (cytoplasmic fraction) was collected. The precipitate was then washed three times by the same mild buffer and dissolved with RIPA buffer to collect the nuclear fraction. Protein concentration was measured by the BCA protein assay kit (Thermo Scientific) and a total of 20 μg protein was loaded for analysis by SDS-PAGE. After the protein transfer, nitrocellulose membranes (BIO-RAD) were blocked with Tris-buffered saline containing 5% Difco Skim Milk (BD) and 0.1% Tween 20 for 1 h at room temperature. Membranes were then incubated at 4°C overnight with the primary antibodies. After treatments with primary antibodies, membranes were washed, and then incubated with horseradish peroxidase-coupled goat anti-mouse or anti-rabbit IgG secondary antibody (Cell Signaling Technology, Boston, MA, United states) at room temperature for 2 h. Specific signals from the transferred protein were then visualized by SuperSignal West Pico Chemiluminescent Substrate (Thermo Scientific) using a LAS4000 biomolecular imager. ImageJ software was used to quantify the signals.

### Chromatin Immunoprecipitation (ChIP) Assays

ChIP assays were conducted as the published protocol (Carey et al., 2009). DNA/protein complexes in culturing cells were cross-linked with 4% paraformaldehyde (PFA) for 20 min at 4°C, and those in brain tissues were cross-linked by the transcardial perfusion of 4% PFA (30 ml/mice). Cells and tissues were then lysed in the lysis buffer (5 mM PIPES of pH8.0, 85 mM KCl, and 0.5% Nonidet P-40 dissolved in dH_2_O). DNA in the DNA/protein complexes was fragmented to 200–400 bp, which correspond to the size of mono-to di-nucleosomes, by sonication in ice-cold water. We then used 1 mg DNA-protein complexes in dilution buffer (16.7 mM Tris, 167 mM NaCl, 1.2 mM EDTA, 0.01% SDS, and 1.1% Triton X-100) for each experimental group, and treated with the protein A-agarose/salmon sperm DNA (Millipore) for 2 h at 4°C with rotation. After centrifuging at 3,000 rpm for 2 min, supernatants were collected into a new tube and rotated with 500 ng of the primary antibodies (p-CREB, 9,197, Cell Signaling) overnight at 4°C. We then added protein A-agarose/salmon sperm DNA to the supernatant and rotated for 2 h followed by centrifugation at 3,000 rpm for 2 min. The precipitated beads were then washed with high-salt wash buffer (50 mM HEPES, 500 mM NaCl, 1 mM EDTA, 0.1% SDS, 1% Triton X-100, and 0.1% deoxycholate) with rotation for 10 min at room temperature followed by centrifugation at 3,000 rpm for 2 min. This high-salt wash was repeated 8 times followed by 2 times wash with the TE buffer (1mM EDTA and 10 mM Tris). The precipitated chromatins were then re-suspended in elution buffer (50 mM Tris pH8.0, 10 mM EDTA and 1% SDS) followed by treatment with Proteinase-K (20 μg/ml) at 55°C for 2 h. To reverse cross-links, samples were then treated at 65°C overnight followed by centrifugation at 12,000 rpm for 5 min. The supernatant was then collected and subjected to DNA purification by standard phenol: chloroform extraction method. Amplification of CREB binding sites mentioned here as responsive elements (RE) onto the mouse *Bdnf* genomic region was carried out using conventional PCR with rTaq DNA polymerase (R001A, Takara) as well as by the real-time PCR assays using the following primer sets: for RE-1, forward 5′-CCT​CCC​ACG​TCA​TTT​TAC​GA-3′ and reverse 5′-AGC​CAG​TTT​CCT​GAG​AAT​GC-3′; for RE-2, forward 5′-CTG​AGC​AAA​GCC​GAA​CTT​CT-3′ and reverse 5′-GCT​TTG​CTG​TCC​TGG​AGA​CT-3′; for RE-3, forward 5′-TCT​GGG​CGA​CAA​GGA​GAA​AA-3′ and reverse 5′-TGG​GTG​TAA​GAG​GAT​GAC​GT-3′, for RE-4, forward 5′-GGG​GTT​GCC​TTA​AGT​GGA​GA-3′ and reverse 5′-GAA​CAG​CTC​TAC​ATT​CCC​AAC​T-3′; for RE-5, forward 5′-CAC​AGT​GAC​TTG​GGT​TCA​AAG​A-3′ and reverse 5′-TGT​GCA​TGG​AAA​CAG​AAA​CTG-3′, for RE-6, forward 5′-GCT​GAA​TTT​ATT​GTA​CAT​GCG​GT-3′ and reverse 5′-CCC​ACA​CTA​ACC​AGC​CTG​AT-3′; for RE-7, forward 5′-TTT​TCC​CCA​AAG​AAG​AGT​GAA​G-3′ and reverse 5′-TTG​GAT​GAG​GTC​AAT​CCT​ACT​ATG-3′, for RE-8, forward 5′-TGT​CTA​TTT​CGA​GGC​AGA​GGA-3′ and reverse 5′-CTC​CTC​GGT​GAA​TGG​GAA​AG-3′; for RE-9, forward 5′-CAA​CGA​CAC​AGA​ACA​CAC​GTT-3′ and reverse 5′-GCA​CAA​CAG​TCT​TGT​TTA​TCT​GG-3′, for RE-10, forward 5′-TGT​AAT​AGA​AAG​TCA​GAT​AAA​TGT​TTC​AA-3′ and reverse 5′-TAC​TGG​GAA​GTC​TGG​GGA​AA-3′; and RE-11, forward 5′-CCT​CTT​GGA​AAG​CAA​CGT​GT-3′ and reverse 5′-TGG​TGG​GAG​ACT​GAC​ATC​AA-3′. For the negative control of ChIP assays, we amplified the intron-region right after the exon-10 (Bdnf-10) by the primers forward 5′-TCA​CCG​TAT​CCG​CTG​CCT-3′ and reverse 5′-CCA​GGT​GGT​GTC​TCA​GAT​AC-3′. The size of the amplified genome by these primer sets was within 100–150 bp.

### Immunohistochemical Staining

To prepare the cryosections, mice were perfused transcardially with phosphate-buffered saline (PBS) followed by 4% PFA solution. The brains were then collected in 4% PFA solution, fixed overnight at 4°C followed by treatment with 30% sucrose solution, embedded in optimum cutting temperature (O. C. T.) compound, and frozen in −70°C before making thin sections (30 μm thick). Sections were collected in ice-cold PBS and treated to 3N HCl for 30 min followed by extensive washing with PBS on a shaker. After 1 h treatment with a blocking solution (0.2% TritonX-100 and 5% BSA dissolved in PBS), tissue slices were incubated with the antibodies, dissolved in the same blocking solution, overnight at 4°C on a shaker. Antibodies were diluted as follows: p-Akt (Cell signaling, 1:500), p-ERK (Cell signaling, 1:500), and DCX (Abcam, 1:500). After brief washing in PBS, the brain slices were then incubated with fluorochrome-conjugated secondary antibodies (1:1,000 in blocking solution; Alexa Fluor 488/568, Life technologies) for 3 h at room temperature. 4′,6-diamidino-2-phenylindole (DAPI) was used for nuclear staining. The images were analyzed by BZ-9000 Fluorescence Microscope (KEYENCE, Osaka, Japan).

### Electrophysiology

The electrophysiology assay of the mice hippocampal slices was performed according to the protocol described in the published paper (Hossain et al., 2018). Under deep anesthesia with isoflurane, mice were sacrificed and the brain was quickly excised. The hippocampal slices (350 μm thick) were made perpendicular to the septotemporal axis and incubated in Krebs-Ringer solution (124 mM NaCl, 4 mM KCl, 1.3 mM MgSO4, 1.23 mM NaH2PO4, 26 mM NaHCO3, 2.4 mM CaCl2, and 10 mM glucose) bubbled with 95% O_2_ and 5% CO_2_ at 32–34°C with pH 7.4. After 2 h incubation, slices were transferred to a recording chamber, which was perfused with Krebs-Ringer solution at a constant rate of about 2.5 ml/min. Extracellular recording of field excitatory postsynaptic potential (EPSP) was made from the stratum radiatum in the area CA1 by a glass microelectrode filled with Krebs-Ringer solution (tip diameter, 15–25 μm; DC resistance, 3–5 MΩ). Orthodromic stimuli were delivered through a coaxial bipolar electrode (diameter, 0.3 mm) that was placed in the stratum radiatum in the CA3 region to stimulate the Schaffer collateral-CA1 pathway. The test-stimulus intensity of 50 µs square pulses ranged from 0.15 to 0.4 mA to give a field EPSP amplitude of 0.2–0.4 mV at 0.03 Hz. After confirmation of stable amplitude of EPSP at least for 10 min, tetanic stimulation was delivered, and data were collected further for 60 min. To induce LTP, a train of 100 Hz for 1 s was delivered at the same stimulus intensity used for the test stimulus. Responses were acquired, digitized, and stored by a Macintosh computer interfaced with PowerLab (AD instruments, New South Wales, Australia) at 20 kHz for 64 ms, beginning 4 ms before the stimulation. For LTP analysis, the initial slope of the EPSP at each time point was expressed as a percentage of control values (averages before tetanic stimulation).

### Golgi Cox Staining

To prepare the Golgi-Cox solution, we first prepared three solutions: solution A (5% potassium dichromate, K_2_Cr_2_O_7_, in distilled water), solution B (5% mercuric chloride, Hg_2_Cl_2_, in dH_2_O), and solution C (5% potassium chromate, K_2_CrO_4_, in dH_2_O). 200 ml of each solution A and B were mixed in a glass bottle followed by adding 160 ml of solution C slowly under continuous agitation by glass rods (special precaution was taken to avoid the skin and inhalation contact and all these mixings were done under fume hood chamber). The mixed solution (Golgi-Cox solution) was kept in the dark for 5 days followed by adding the intracardiac saline perfused mice brains. After 14 days in the solution, the brains were kept in 30% sucrose solution overnight followed by freezing at −70°C with the O.C.T. compound (Tissue-Tek). The frozen brain tissues were then cut into 30 μm slices and collected on FRONTIER coated glass slides (FRC-03, Matsunami Glass Ind. Ltd., Osaka, Japan) followed by stock in −70 °C. Before use, the slides were kept in 70% EtOH for 3 min followed by drying at room temperature aimed to improve the tissue adhesion. The slides were then subjected to dH_2_O for 1 min followed by 30 min treatments with 10% ammonium hydroxide solution in a dark room. Slides were then washed in dH_2_O for 1 min followed by 30min treatments with Kodak Fix Solution (251 ml of Kodak Fix Solution A and 28 ml of Kodak Fix Solution B in 2000ml dH_2_O). Slides were washed in dH2O for 1 min followed by the alcohol dehydration process: 30 s in 70% ethanol, 30 s in 80% EtOH, 30 s in 90% ethanol, 30 s in 95% ethanol, and 2 min in 100% ethanol (2 times). Before mounting with the Permafluor (Thermo scientific) the slides were treated with CXA (Chloroform, Xylene, and Alcohol in a ratio of 1:1:1) for 15 min. Photographs were taken by the BZ-9000 Fluorescence Microscope by using the bright field filter.

### Lipid Raft Isolation and Cholesterol Assays

Brain tissues were dissolved in the raft buffer composed of 100 mM HEPES, 5 M NaCl, and 50 mM EGTA by passing through 25 G needles 15 times. We then added 1% Brij-58 (P5884, Sigma) and passed through 25 G needles for 15 times followed by keeping the solution in Ice for 30 min. A total of 1 mg protein was then dissolved in sucrose-PBS solution to make 4 ml volume of 40% sucrose mixture followed by adding it in the centrifuge tube (13PET TUBE ASSY, S303276A, Hitachi, Japan) keeping on ice. We then slowly added 4 ml of 25% sucrose-PBS solution followed by adding 4 ml of 5% sucrose-PBS solution to make a total of 12 ml solution in the tube. The centrifuge tubes were then placed in the ultracentrifuge machine (himac CP 65β, Hitachi Centrifuges, Japan) at a speed of 36,000 rpm for 26 h at 4°C. After the centrifuge, 12 fractions of 1ml volume were collected gently from top to the bottom and labeled fractions No. 1–12. A total of 250 μL 59% (w/v) trichloroacetic acid (TCA) solution was added to each 1 ml fraction and mixed vigorously followed by centrifugation at 14,000 rpm for 10 min. The protein precipitates were then washed with acetone 3 times followed by being subjected to the heat block at 95°C until dried up. SDS sample buffer (50 μL) was used to dissolve the protein before performing Western blotting. The cholesterol contents of the lipid raft fractions were analyzed by the total cholesterol assay kit (Cell Biolabs, Inc. Catalog number STA-384) by following the recommended protocol.

### Liquid Chromatography-Electronspray Ionization-Tandem Mass Spectrometry (LC-MS) Assays

Extraction of the total lipids was performed by Bligh and Dyer method (Bligh and Dyer, 1959; Abe et al., 2014). Briefly, the cells and tissues were dissolved in PBS followed by sonication in the ice-cold water. After checking the protein concentration, equal amount (50 μg) of protein was dissolved in methanol/chloroform/water (v/v/v: 2:1:0.8) and 50 pmol internal standard (1-heptadecanoylsn-glycero-3-phosphocholine, 1, 2-didodecanoyl-sn-glycero-3-phosphocholine and 1, 2-didodecanoyl-sn-glycero-3-phosphoethanolamine). After 5 min, 1 ml each of water and chloroform was added and the whole centrifuged to collect the lower organic phase. 1 ml chloroform was added again to re-extract the lipids. Collected organic phase was then evaporated under the nitrogen stream and suspended in pure methanol. LC-MS assay was performed using a 4000 Q-TRAP quadrupole linear ion trap hybrid mass Q8 spectrometer (AB Sciex) with ACQUITY UPLC System (Waters). Samples were injected into an ACQUIRY UPLC BEH C18 column and then directly subjected to ESI-MS/MS analysis. A 10 μL aliquot of each sample was directly introduced by autosampler injector and the samples were separated by step gradient elution with mobile phase A (acetonitrile:methanol:water at 2:2:1 (v/v/v), 0.1% formic acid and 0.028% ammonium) and mobile phase B (isopropanol, 0.1% formic acid and 0.028% ammonium) at the ratios: 100:0 (for 0–5 min), 95:5 (5–20 min), 70:30 (20–21 min), 50:50 (21–45 min), 50:50 (45–100 min) and 100:0 (100–120 min). The flow rate (70 μL/min at 30°C), source temperature (200°C), declustering potential (60), and collision cell exit potential (15) was kept constant. Ethanolamine Pls (PlsEtns) with alkenyl p16:0, p18:0, and p18:1 at the sn-1 position was detected by precursor ion scan of m/z 364, m/z 392, and m/z 390, respectively, at positive ion mode. The data were analyzed and quantified using the Analyst software (AB Sciex).

### Statistical Analyses

All data were expressed as mean ± S.E.M (standard error of mean). To analyze the statistical significance among experimental groups, we performed one-way ANOVA followed by a post hoc test (Bonferroni’s test). Student’s t-tests were performed to find the significance values in the case of two experimental groups. *p* values less than 0.05 were considered significant.

## Data Availability

The original contributions presented in the study are included in the article/Supplementary Material, further inquiries can be directed to the corresponding author.
