# Supplementary figures and images for: Plasmalogens, the Vinyl Ether-Linked Glycerophospholipids, Enhance Learning and Memory by Regulating Brain-Derived Neurotrophic Factor
Source: Front Cell Dev Biol. 2022 Feb 9;10:828282. doi: 10.3389/fcell.2022.828282 (PMC8864319; doi:10.3389/fcell.2022.828282)

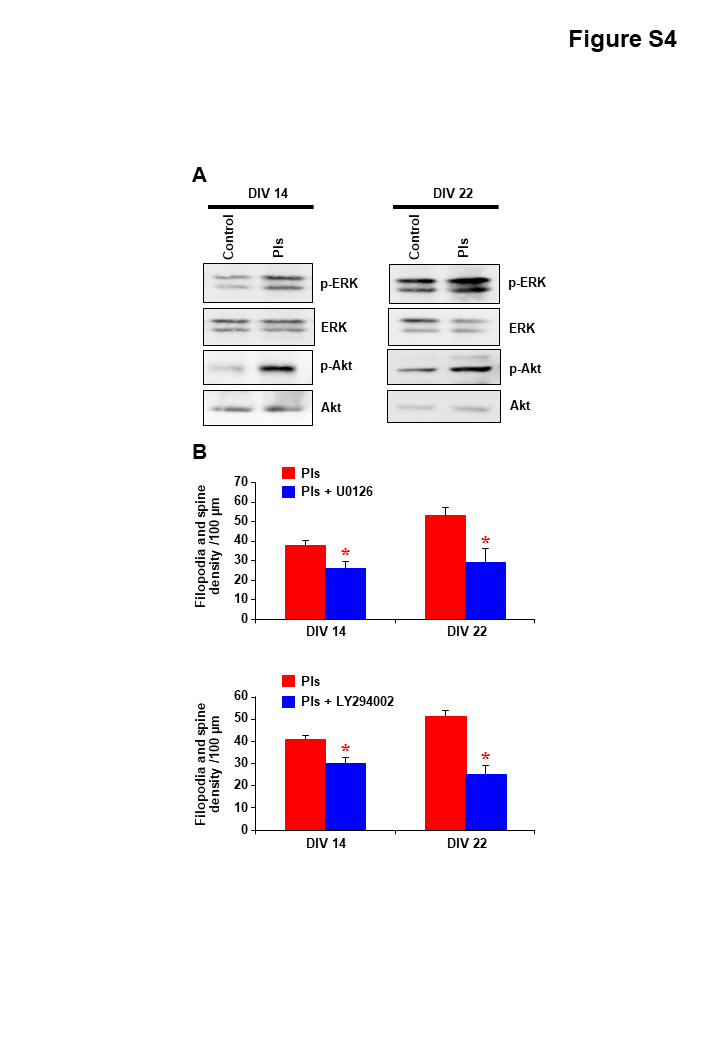

Supplement: Supplementary file 1 [file Image6.TIF]

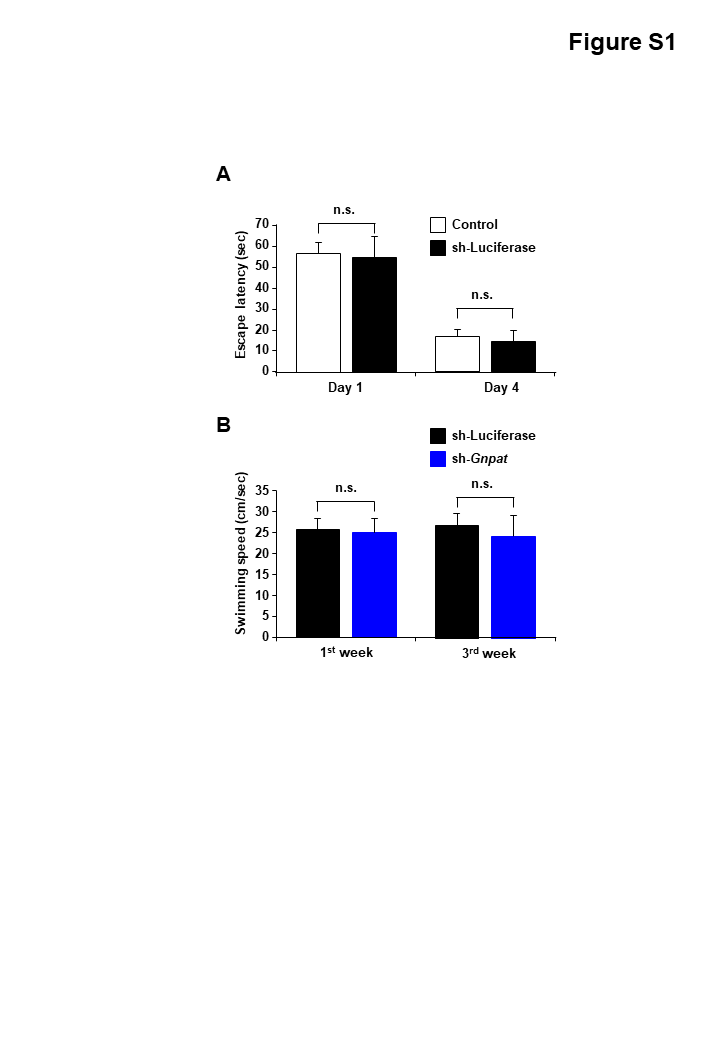

Supplement: Supplementary file 2 [file Image3.TIF]

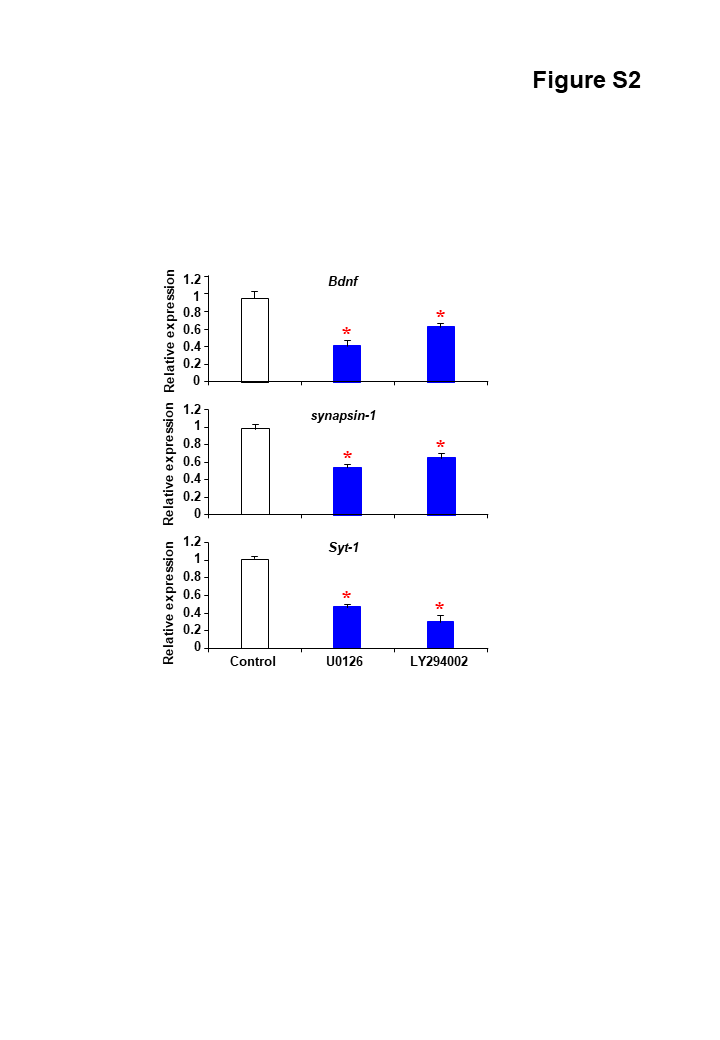

Supplement: Supplementary file 3 [file Image4.TIF]

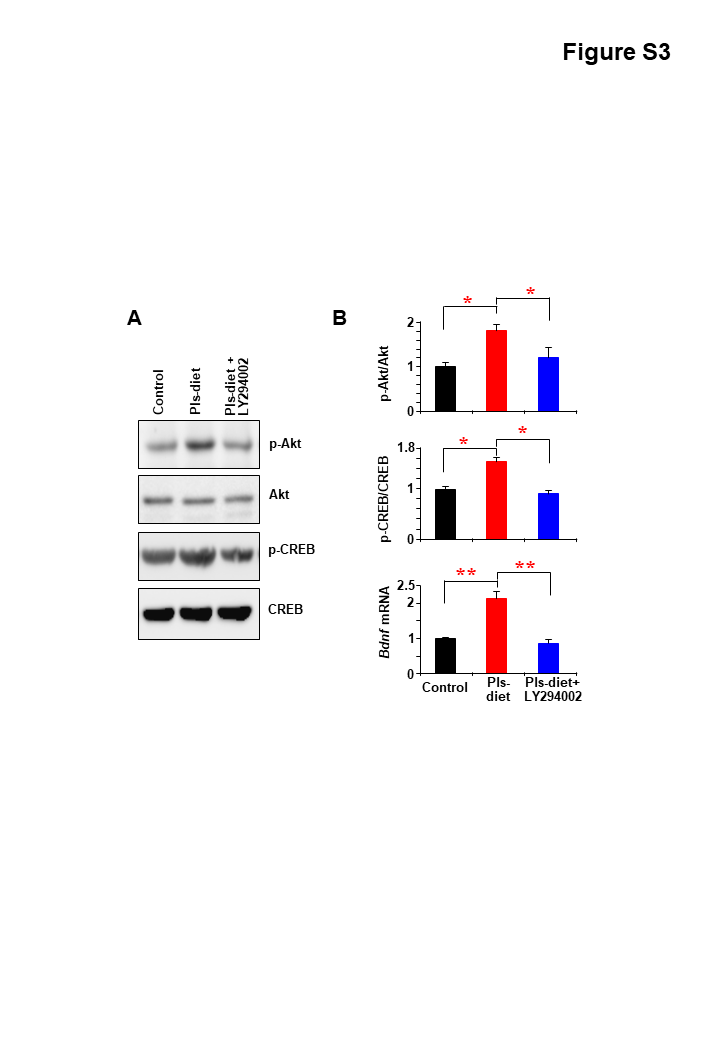

Supplement: Supplementary file 4 [file Image5.TIF]
