# Supplementary material for: Plasmalogens, the Vinyl Ether-Linked Glycerophospholipids, Enhance Learning and Memory by Regulating Brain-Derived Neurotrophic Factor
Source: Front Cell Dev Biol. 2022 Feb 9;10:828282. doi: 10.3389/fcell.2022.828282 (PMC8864319; doi:10.3389/fcell.2022.828282)
Supplement: Supplementary file 5 [file DataSheet1.docx]

**Supplementary Fig S1. No change in the memory and swimming speed by the lentivirus injection. A**. Mice were injected with the control lentiviruses and compared the memory task with that of the control mice with no surgical operation. Mice were subjected to the memory task as in Fig 1 and the escape latency was checked on day 4. There were no differences in memory between the groups. **B.** The swimming speed was checked among the two groups of mice as in Fig 1. No changes in the swimming speed were detected. The speed was checked through all the tasks. The data represent mean ± S.E.M. (n = 5). The P values were calculated by Student’s t-test.

**Supplementary Fig S2. Reduction of hippocampal *BDNF*, *synapsin-1,* and *SYT-1* mRNAs by ERK and PI3K/Akt inhibitors.** ERK (U0126) or PI3K/Akt inhibitor (LY294002) was stereotaxically injected into the bilateral hippocampus (0.5 µl of 50 µM solution/site). Real-time PCR analysis performed 24 hr after the injection showed the relative reduction of hippocampal *Bdnf*, *synapsin-1,* and *Syt-1* mRNAs in normal mice. The data represent mean ± S.E.M. (n=7, ***,** *P*<0.05, ANOVA followed by Bonferroni’s post hoc tests).

**Supplementary Fig S3.** **Suppression of Pls-induced expression of hippocampal p-Akt, p-CREB, and *BDNF* mRNA by local injection of PI3K/Akt inhibitor (LY294002, 0.5 µl of 50 µM solution/site).** **A.** Representative Western blotting assays show hippocampal expressions of p-Akt, total Akt, p-CREB, and total CREB proteins in mice given control diet, Pls diet, and Pls diet with a hippocampal injection of PI3K/Akt inhibitor. Western blotting analysis was performed 24 hr after the injection. **B.** Quantification data of the panel (**A**) and real-time PCR analysis show that the Pls-induced increases in p-Akt, p-CREB, and *Bdnf* mRNA are suppressed by hippocampal injection of PI3K/Akt inhibitor. Each experiment included more than 5 mice. The data represent mean ± S.E.M. *, *P*<0.05 and **, *P*<0.01 (ANOVA followed by Bonferroni’s post hoc tests to compare between the groups).

**Supplementary Figure 4. Involvement of ERK and Akt activation in Pls-induced spine densities.** **A.** Western blotting data show the increased expression of phosphorylated (p-) ERK and Akt in the Pls (5 µg/ml)-treated primary neuronal cells on DIV 14 and DIV 22. Pls were added on the DIV3. **B.** Number of dendritic filopodia and spines decreased by ERK (U0126) and PI3K/Akt (LY294002) inhibitors (5 µM, respectively), which were added 24 hr before counting. The data represent mean ± S.E.M. (n = 3, ***,** *P*<0.05, Student’s t-test).
